# Dynamic Labeling Reveals Temporal Changes in Carbon Re-Allocation within the Central Metabolism of Developing Apple Fruit

**DOI:** 10.3389/fpls.2017.01785

**Published:** 2017-10-18

**Authors:** Wasiye F. Beshir, Victor B. M. Mbong, Maarten L. A. T. M. Hertog, Annemie H. Geeraerd, Wim Van den Ende, Bart M. Nicolaï

**Affiliations:** ^1^Division of Mechatronics, Biostatistics and Sensors, Department of Biosystems, KU Leuven, Leuven, Belgium; ^2^Laboratory of Molecular Plant Biology, Department of Biology, KU Leuven, Leuven, Belgium; ^3^Flanders Centre of Postharvest Technology, Leuven, Belgium

**Keywords:** *Malus domestica* Borkh, Braeburn, fruit growth, GC-MS, metabolomics, ^13^C-label accumulation

## Abstract

In recent years, the application of isotopically labeled substrates has received extensive attention in plant physiology. Measuring the propagation of the label through metabolic networks may provide information on carbon allocation in sink fruit during fruit development. In this research, gas chromatography coupled to mass spectrometry based metabolite profiling was used to characterize the changing metabolic pool sizes in developing apple fruit at five growth stages (30, 58, 93, 121, and 149 days after full bloom) using ^13^C-isotope feeding experiments on hypanthium tissue discs. Following the feeding of [U-^13^C]glucose, the ^13^C-label was incorporated into the various metabolites to different degrees depending on incubation time, metabolic pathway activity, and growth stage. Evidence is presented that early in fruit development the utilization of the imported sugars was faster than in later developmental stages, likely to supply the energy and carbon skeletons required for cell division and fruit growth. The declined ^13^C-incorporation into various metabolites during growth and maturation can be associated with the reduced metabolic activity, as mirrored by the respiratory rate. Moreover, the concentration of fructose and sucrose increased during fruit development, whereas concentrations of most amino and organic acids and polyphenols declined. In general, this study showed that the imported compounds play a central role not only in carbohydrate metabolism, but also in the biosynthesis of amino acid and related protein synthesis and secondary metabolites at the early stage of fruit development.

## Introduction

Apple (*Malus domestica* Borkh.) is a member of the *Rosaceae* family that includes many important fruit trees like pear, peach, cherry, apricot, and prune. Apple is the most important fruit in the world market followed by pear and peach (Brown, 2012). It is consumed widely for its flavor, health, and nutritional value. The composition of the mature apple fruit is the resultant of carbohydrate import and the metabolic processes occurring during fruit development.

**Figure 1:**
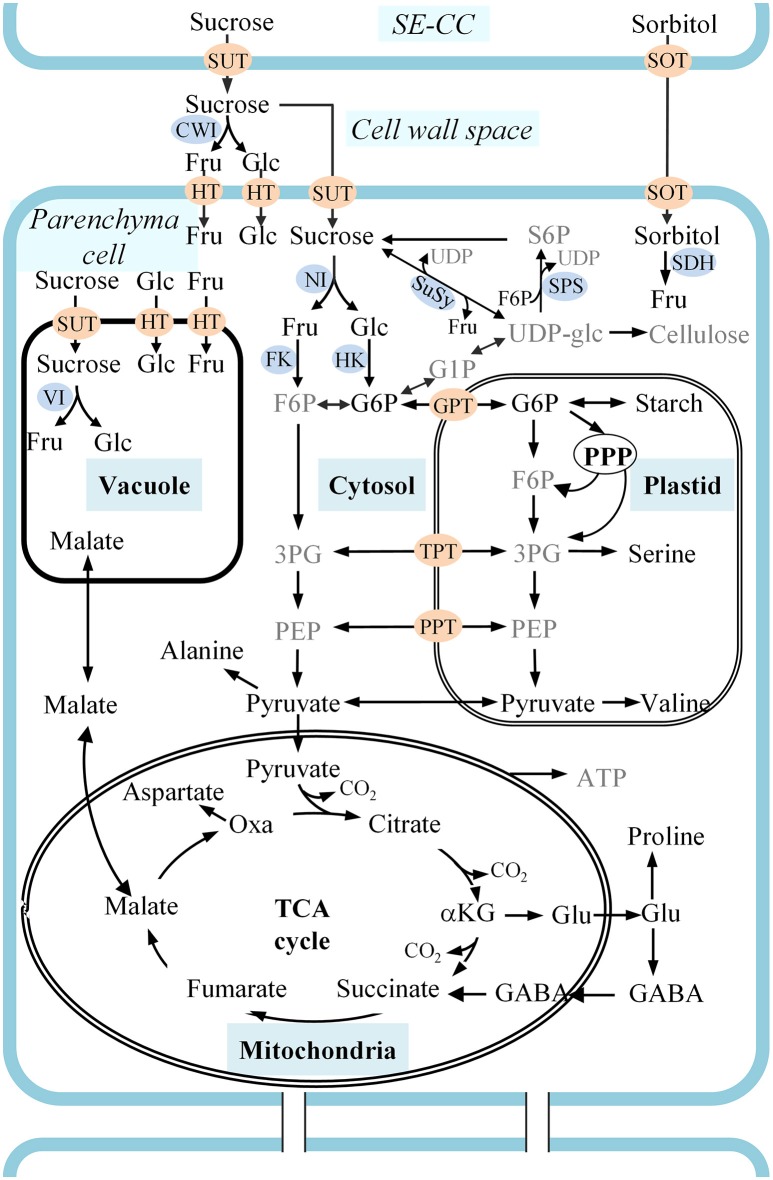
A schematic representation of carbon metabolism in developing apple fruit. Sorbitol and sucrose are transported from the leaves to the fruit and unloaded in the cell wall space between sieve element-companion cell complexes (SE-CC) and parenchyma cells. Sorbitol is transported from the cell wall space into cells by sorbitol transporters (SOT). Sucrose is either directly transported into cells by sucrose transporters (SUT), or is converted into fructose and glucose in the cell wall space by cell wall bound invertase (CWI) before being transported into the cells by hexose transporters (HT). Metabolic pathways are described considering carbon metabolism in developing apple fruit and using the labeling of intermediates described in the text and information in the literature. Metabolites presented in gray text are those whose ^13^C-labeling could not be reliably quantified in this study. αKG, α-ketoglutarate; 3PG, 3-phosphoglycerate; F6P, fructose 6-phosphate; Fru, fructose; G1P, Glucose 1-phosphate; G6P, Glucose 6-phosphate; GABA, γ-aminobutyrate; Glc, glucose; GPT, G6P/phosphate transporter; NI, neutral invertase; Oxa, oxaloacetate; PEP, phosphoenolpyruvate; PPP, pentose phosphate pathway; PPT, PEP/phosphate transporter; S6P, sucrose 6-phosphate; TCA cycle, tricarboxylic acid cycle; UDP-glc, uridine diphosphate-glucose; VI, vacuolar invertase; XPT, triose-phosphate transporter.

In plants, carbon is usually exchanged between source and sink tissues as simple sugars, typically sucrose (White et al., 2015). However, in *Rosaceae*, sorbitol and sucrose are the two main photosynthates, comprising, for instance, respectively 70 and 30% of the components collected from phloem exudate of apple fruit stalks (Klages et al., 2001). Phloem unloading of soluble sugars in developing apple and pear fruit follows an apoplastic route (Zhang et al., 2004, 2014) with sorbitol and sugar transporters being responsible for the uptake by the cells (Watari et al., 2004; Fan et al., 2009; Peng et al., 2011). Sorbitol is converted into fructose by different types of sorbitol dehydrogenase (SDH) enzymes (Loescher et al., 1982) showing different subcellular localizations in the different tissue types of apple (Wang et al., 2009), perhaps indicating its distinct role in the different tissues. Sucrose is either directly transported into parenchyma cells by sucrose transporters (SUT), or is converted into fructose and glucose in the apoplast by cell wall bound invertase (CWI) before being transported into the cells by hexose transporters (HT) (Büttner and Sauer, 2000; Williams et al., 2000). Subsequently, the imported compounds enter the fruit's respiration pathway to generate the energy to fuel metabolic processes (White et al., 2015) and to contribute to sucrose, fructose, glucose, malate, and starch pools (Berüter et al., 1997; Li et al., 2012) (see Figure 1).

In the field of systems biology there is a growing interest in using “omics” technologies, mainly genomics, transcriptomics, proteomics, and metabolomics for better understanding growth and ripening related changes of apple fruit (Janssen et al., 2008; Zhang et al., 2010; Henry-Kirk et al., 2012; Li et al., 2012). Metabolomics plays a central role in systems biology focusing on identification and quantification of low molecular weight metabolites, which are end products of cellular regulation, especially when encountering various stress conditions (Fiehn, 2002; Fernie et al., 2004b; Roessner and Beckles, 2009). Metabolomics studies are commonly used to characterize complex physiological and biochemical changes occurring during fruit development (Zhang et al., 2010; Li et al., 2013). Nonetheless, knowledge of metabolite levels by itself is insufficient to unravel intracellular fluxes related with activity levels in different pathway and regulatory mechanisms. For example, fluxes through a pathway can change without a significant change in the levels of intermediate metabolites (Fernie et al., 2005). The application of isotopically labeled substrates to reveal the *in vivo* carbon flow levels through metabolic networks in responses to physiological stimuli or genetic modification has, lately, received extensive attention (Schwender et al., 2004; Sauer, 2006; Ampofo-Asiama et al., 2014; Buescher et al., 2015; Heux et al., 2017; Mbong et al., 2017a,b). Previous isotope labeling studies in developing apple fruit were focused at a specific growth stage (Berüter et al., 1997; Berüter, 2004) rather than covering the journey from flowering to fully mature fruit. This has triggered the question of how the metabolite levels and metabolic pathway activity changed with the various stages of apple fruit development, covering cell division, cell expansion, and maturation.

Therefore, the aim of this study was to create a comprehensive understanding of the dynamics of metabolic changes occurring throughout apple fruit development by studying the changing uptake and distribution of ^13^C-label through feeding experiments on hypanthium tissue discs taken at distinct stages of fruit development. It is the first time that dynamic isotope labeling experiments have been performed at various stages of apple fruit development to study changes in carbon re-allocation during fruit growth. A wide range of polar metabolites were analyzed using gas chromatography coupled to mass spectrometry (GC-MS) characterizing the metabolic pool sizes and the ^13^C-label distribution in growing apple fruit at distinct stages of development covering all major events occurring during fruit development.

## Materials and methods

### Plant materials and chemicals

Apples (*Malus domestica* Borkh., cv. “Braeburn”) collected from seven designated 2-year-old trees grown at the KU Leuven Research orchard at Rillaar, Belgium (50°57′48.8″N, 4°52′56.5″E) were used for this study. During the 2015–2016 growing season fruit samples were harvested at five growth stages (30, 58, 93, 121, and 149 days after full bloom, i.e., after the flowering is fully completed, see Figure S1). Fruits were harvested in the morning and immediately transported to the MeBioS lab, KU Leuven, where the experiments were carried out. At each stage, at least 45 replicate fruits were collected from the seven trees.

Previously published physiological and morphological data from apple fruit development (Janssen et al., 2008; Li et al., 2012) were used to select stages matching five major events occurring during fruit growth and development, i.e., 30 days (cell division), 58 days (peak rate of cell expansion and starch accumulation), 93 days (decline of cell expansion rate), 121 days (decline of starch levels), and 149 days (ripening) after full bloom.

Analytical grade chemicals were purchased from Sigma-Aldrich (Belgium) and stored according to the manufacturer's instructions.

### Respiration, ethylene production, and osmolality measurements

The results used to characterize the physiological parameters of developing apple fruit were all expressed on a fresh weight basis. The respiration and ethylene production rates of developing apple fruit were measured based on the methods described in Bulens et al. (2011). With respect to the five growth stages, one or two fruits were placed in 1-liter air tight glass jars. The jars were flushed with regular air. After 2 h of flushing, the gas flow was stopped and the initial composition of the gases in the headspace was measured using a compact-GC (Interscience, Louvain La Neuve, Belgium). The final reading was taken after 24 h. Respiration and ethylene production rate were calculated from these data with respiration rate being expressed in terms of CO_2_ production rate (nmol kg^−1^ s^−1^).

The osmolality of the fruit was determined based on the freezing point depression of pressed juice extract of 10 fruits, as described previously (Berüter, 2004). After the water activity of the extracted juice was measured using aw-Kryometer (AWK-40; Nagy, Germany), the osmolality of the fruit was derived from a calibration curve prepared using NaCl_2_ solutions of known osmolality.

### ^13^C-isotope labeling experiments

*In vivo* ^13^C labeling experiments were conducted using apple tissue discs cut from the harvested apple fruit, submerged in a liquid medium supplemented with 20 mM [U-^13^C]glucose (Figure S1). The choice of [U-^13^C]glucose was based on the assumption that glucose is transported into the parenchyma cells after sucrose being converted into glucose and fructose by apoplastic CWI. The fruit was sliced along the equatorial axis and tissue discs (~10 mm diameter and ~1 mm thickness) were collected from the hypanthium tissue using a cork borer. Excised tissue discs were washed three times in an isotonic solution containing 50 mM HEPES/KOH buffer (pH 7.0), 2 mM CaCl_2_, 2 mM MgCl_2_, 2 mM DTT (1,4-dithiothreitol) using betaine as an osmoticum, and while shaking at 90 rpm, to remove damaged cells. The osmotic strength of the solution was adjusted to the osmolality of the different growth stages to preserve the integrity of tissue discs submerged in liquid medium. As a result, the betaine concentration in the medium was increased from 160 mM at 30 days to 730 mM at 149 days. After washing thoroughly about 2.5 g of tissue discs was placed in a 50 ml flask containing 5 ml of buffer solution. This solution was identical to the one used to prepare the tissue discs, yet supplemented with 20 mM unlabeled glucose. After pre-incubation in unlabeled solution for 12 h the tissue discs were transferred to a solution containing [U-^13^C]glucose (≥99% enrichment). The solution was continuously aerated with humidified air at 20°C in a controlled environment. Tissue discs were incubated for various time intervals (1, 2, 4, 6, 8, 10, or 24 h) after label introduction. Subsequently, samples were washed three times using 100 ml of a hypotonic solution of 50 mM HEPES/KOH buffer (pH 7.0) to remove the salt and excess of substrate from the tissue. After washing, excess medium was removed from the discs using paper tissue. The tissue samples were rapidly frozen in liquid N_2_ and subsequently stored at −80°C prior to GC-MS analysis. Each ^13^C labeling experiment was performed three times starting from independent biological plant material.

### Primary metabolite and starch analysis

Extraction and derivatization of polar metabolites were performed following the protocols described by Bekele et al. (2014). The frozen hypanthium tissue samples were homogenized using Mixer Mill (Retsch, MM 200, Haan, Germany) at a frequency of 30 Hz for 1 min. 200 mg of fresh weight of powdered frozen apple tissue material was extracted using 1 ml of methanol and incubated in a thermomixer (Eppendorf AG, Hamburg, Germany) at 70°C for 15 min, shaking at 1,400 rpm. The methanol extract was centrifuged at 22,000 g for 20 min at 4°C to separate the aliquot from the pellet. Next, the supernatant aliquot was dried at 50°C on a heating block (Stuart, sample concentrator (SBH CON/1), Bibby Scientific Limited Stone, and Staffordshire, UK) under a stream of nitrogen gas. Subsequently, metabolites were derivatized by methoxymation followed by silylation. Firstly, 120 μl of methoxyamine hydrochloride (20 mg methoxyamine hydrochloride (MEOX) in 1 ml pyridine) was added to each sample and incubated in the thermomixer for 60 min at 30°C. Secondly, 120 μl of BSTFA (N,O-Bis(trimethylsilyl(TMS))trifluoroacetamide) was added to each sample and incubated in the thermomixer for 120 min at 45°C. Finally, 100 μl of the derivatized sample was transferred into glass vials containing deactivated glass inserts. In addition, standard compounds were injected alongside the samples in order to allow for the calculation of absolute metabolite concentrations.

GC-MS analysis was performed on a GC 7890A coupled with 5975C MS (Agilent Technologies, Palo Alto, CA). Initially, the sample was volatilized at 230°C, inside the deactivated glass liner (SGE Analytical Science, Victoria, Australia). The chromatographic separation was performed on HP-5 ms column (30 m × 250 μm ID, 0.25 μm film thickness, Supelco, Bellefonte, CA) with programmed temperature ramp. Helium was used as a carrier gas applying a constant flow of 1 ml min^−1^. Each sample was injected in two different split modes. One with split ratio of 5:1 injection, which was optimized for less abundant metabolites. For this injection, the oven temperature was set to 50°C for 2 min, ramped at 10°C min^−1^ to 325°C and held for 5 min at 325°C (34.5 min run time). Second, a high split ratio of 500:1 was used for most abundant metabolites corresponding to malate, asparagine, quinate, fructose, glucose, sorbitol, and sucrose. In 500:1 split mode, the oven temperature was set to 90°C for 2 min and ramped to 325°C switching between 50 and 10°C min^−1^ and held at 325°C for a further 5 min resulting in a run time of 17.3 min. The mass selective detector (MSD) was operated in the electron ionization (70 eV) mode with quadrupole and MS ion source temperatures maintained at 150 and 230°C, respectively. The detector was activated to record throughout the mass spectra range 35–500 *m/z*.

Starch extraction and determination was carried out using the method described by Hendriks et al. (2003). The pellet obtained after the extraction of polar metabolites was further extracted with 80% ethanol at 80°C for 15 min. The pellets were dried and homogenized in 0.2 mM KOH and incubated for 1 h by heating them at 95°C. After acidification to pH 4.9 with 1 M acetic acid/sodium-acetate buffer, the suspension was digested overnight with a mixture of amyloglucosidase and α-amylase. The glucose content of the supernatant was then used to assess the starch content of the sample.

### Metabolite annotation and ^13^C-label enrichments

Metabolite annotation was performed using Agilent MSD Chemstation Software (Agilent Technologies, Santa Clara, USA) by comparing the acquired spectra with an in-house built library, with the Agilent Fiehn Metabolomics Library, and with the NIST2011 Library (National Institute of Standards and Technology, Gaithersburg, MD, USA) (Table S1). MS Correction Tool was used to correct isotopomer fractions of a particular metabolite fragment for the natural stable isotopes (Wahl et al., 2004). The percentage ^13^C enrichment was calculated from the total abundance of ^12^C and ^13^C ions in a particular metabolite pool (Araújo et al., 2014).

### Data analysis

To reveal the correlation structure of the data principal component analysis (PCA) was conducted using the Unscrambler® X software (version 10.3, CAMO A/S, Trondheim, Norway). Heat maps were generated to compare the concentration of metabolites during fruit development (30, 58, 93, 121, and 149 days after full bloom) using the MultiExperiment Viewer software (MeV v4.9.0, http://www.tm4.org/, Saeed et al., 2003). A paired *t*-test analysis was used to compare the mean difference between the glucosyl and fructosyl moieties of sucrose with a significance level of *p*-value = 0.05 using JMP software, version 13.0 (SAS Institute Inc., Cary, NC, USA).

## Results

### Experimental setup and physiological parameters of developing apple fruit

The results reported in this article were carried out at five stages of fruit development (30, 58, 93, 121, and 149 days after full bloom) throughout the 2015–2016 growing season. Preliminary ^13^C labeling experiments were performed to test the uptake capacity of [U-^13^C]glucose by tissue discs cut from developing apple fruit at three distinct growth stages (from the earlier growing season, 2014–2015) including fully ripe apple fruit. The latter showed little to no ^13^C-label incorporation (Figure S2). In contrast, an appreciable amount of ^13^C-label was incorporated into tissue from developing apple fruit with the percentage labeling varying with progressing fruit development (Figure S2). The percentage labeling of glucose increased with fruit development while fully ripe apple fruit showed the lowest uptake rate. In addition, the incorporation of ^13^C-label into the various metabolites increased linearly with the concentration of [U-^13^C]glucose increasing from 5 to 20 mM (Figure S3).

**Figure 2:**
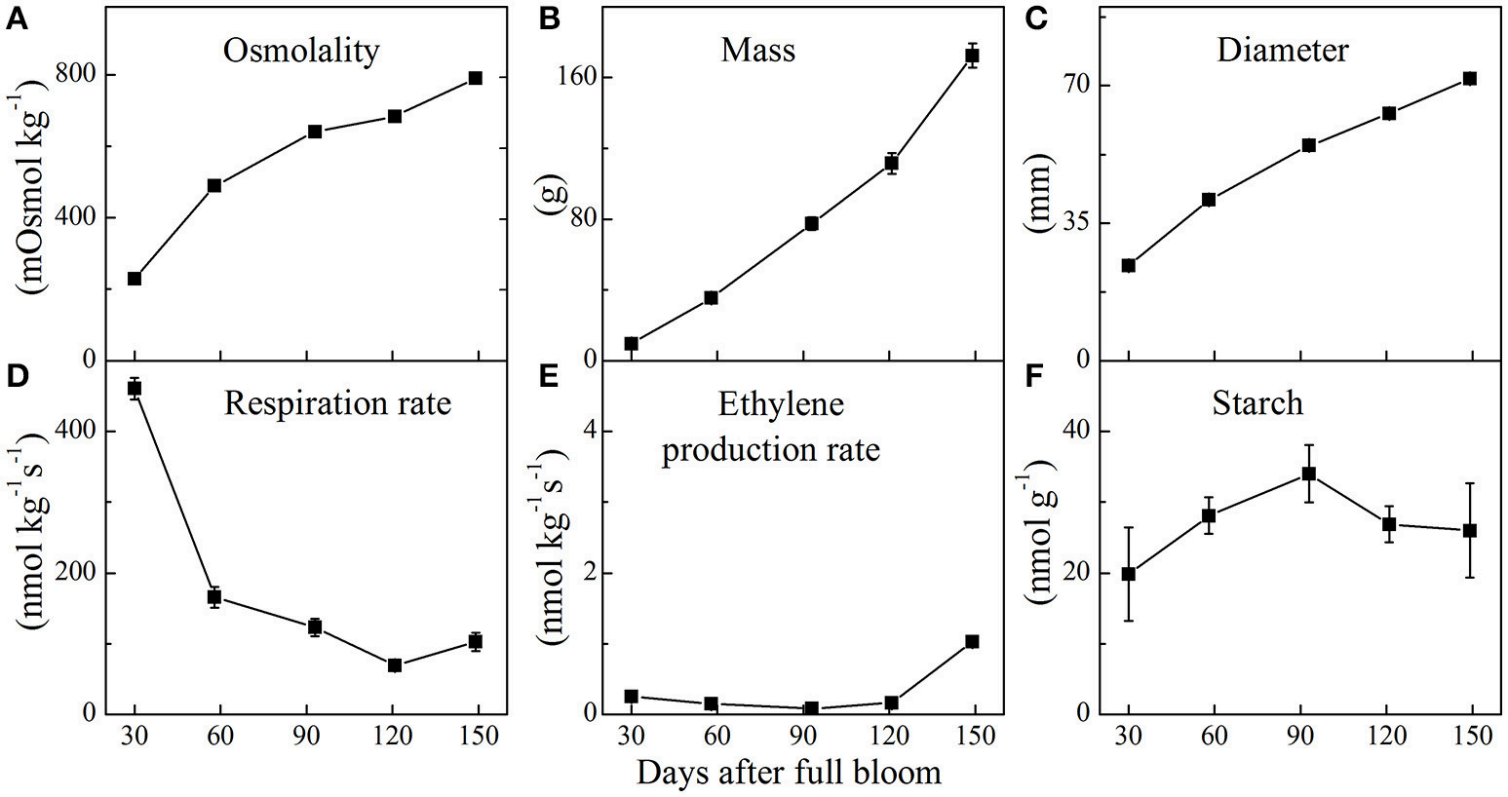
Fruit osmolality **(A)**, mass **(B)**, diameter **(C)**, respiration rate **(D)**, ethylene production rate **(E)**, and starch content **(F)** of “Braeburn” apple throughout fruit development (mean ± SE; *n* = 15). The fruit was sampled at five growth stages throughout the 2015–2016 growing season.

Physiological parameters of apple fruit were measured during fruit development. The changes in fruit osmolality, mass, diameter, respiration and ethylene production rate, and starch content during fruit growth are shown in Figure 2. The osmolality of the fruit increased from 30 days all the way to fruit maturation. Simultaneously, fruit mass and diameter increased approximately linearly from 30 to 149 days. During fruit growth, the respiration rate decreased from 460 nmol kg^−1^ s^−1^ in young fruit to 70 nmol kg^−1^ s^−1^ at 121 days followed by a slight increase at 149 days. In contrast, ethylene production rate remained relatively low until 121 days, showing a slight increase at 149 days. Starch content reached its peak level within 93 days and decreased throughout the later stages of fruit growth.

### Metabolite changes throughout fruit development

**Figure 3:**
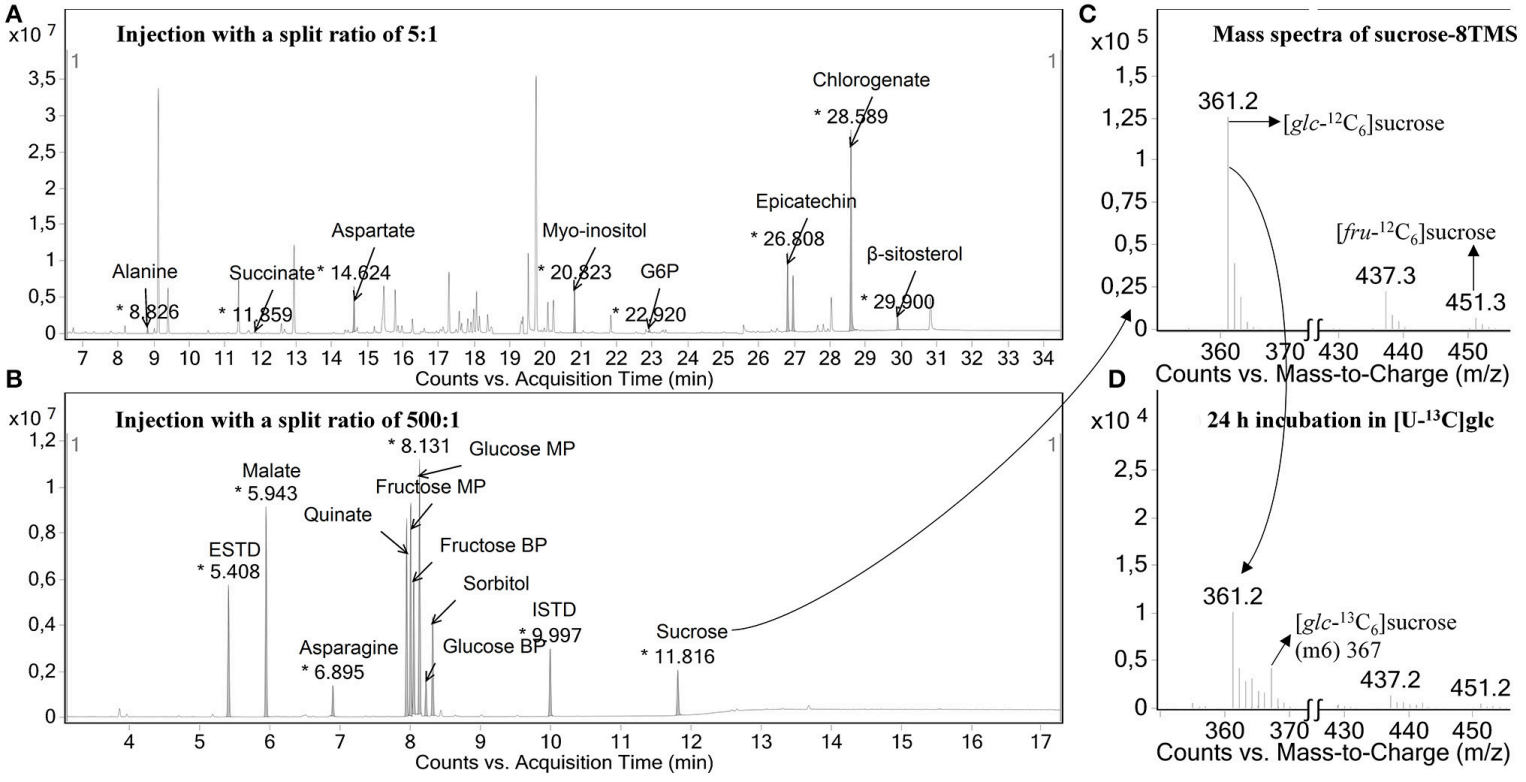
Typical chromatographic results. **(A)** Example of a GC-chromatogram of samples injected with a split ratio of 5:1, which was optimized for less abundant metabolites (34.5 min run time) and **(B)** one with a split ratio of 500:1 used for most abundant metabolites in apple fruit (17.3 min run time). In the former injection mode, the detector was turned off during the time windows where highly abundant peaks were eluting so that those metabolites can be analyzed in a separate injection with a higher split ratio of 500:1 from the same sample. In addition, a mass spectrum generated for TMS-derivatized sucrose before **(C)** and after **(D)** the tissue discs were fed with 20 mM [U-^13^C]glucose are shown. The fragments with *m/z* 361 and *m/z* 451 represent the mass isotopomers of the glucosyl and fructosyl moieties of sucrose, respectively. MP, main product; BP, by product.

The metabolite changes of the developing apple fruit were analyzed using a GC-MS-based metabolite profiling approach. Typical chromatograms are shown in Figure 3. Two injections were realized in this study, one with a split ratio of 5:1 (Figure 3A), which was optimized for less abundant metabolites (34.5 min run time) and one with a split ratio of 500:1 (Figure 3B) used for the most abundant metabolites (17.3 min run time). To verify the identity of the metabolites individual standard compounds were injected. Detailed peak information for the metabolites identified are presented in Table S1.

**Figure 4:**
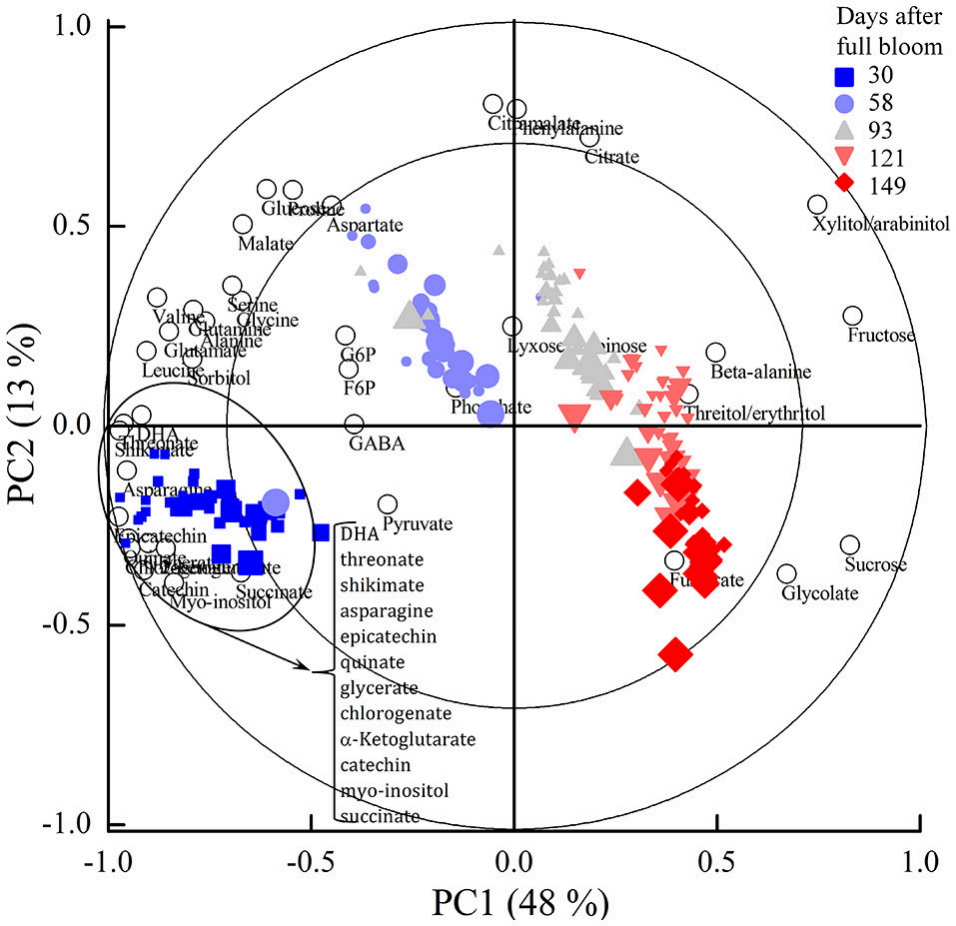
PCA bi-plot showing variation in metabolite levels in relation to the five growth stages and 24 h incubation period with 20 mM [U-13C]glucose of “Braeburn” apple fruit. The loadings of the PCA (open circles) represent individual metabolites. The scores (closed symbols) represent individual samples from the five growth stages (indicated by different colors). The size of the closed symbols reflects the length of the ^13^C-feeding period at each growth stage. The percentage of the explained variances are shown on the axes. PC, principal component.

Figure 4 shows the PCA bi-plot that reveals associations among sugars, sugar alcohols, organic and amino acids, and polyphenols observed in the ^13^C labeling experiments executed at each of the five growth stages. The measured metabolites are represented by the open circles whereas the individual tissue samples are represented by their scores (closed symbols) and colored according to the five growth stages. The amount of variation covered by PC1 and PC2 was 48 and 13%, respectively, covering 61% of the total variance. The scores showed a clear order according to growth stage. The largest variation was observed between the five growth stages (30, 58, 93, 121, and 149 days) whereas the effect of changes in incubation time was less pronounced (as indicated by the size of the closed symbols). Based on the PCA bi-plot some of the sugars, such as fructose and sucrose were positively associated with progressing growth stage. In contrast, most of the organic and amino acids and polyphenols were negatively correlated with growth stage.

**Figure 5:**
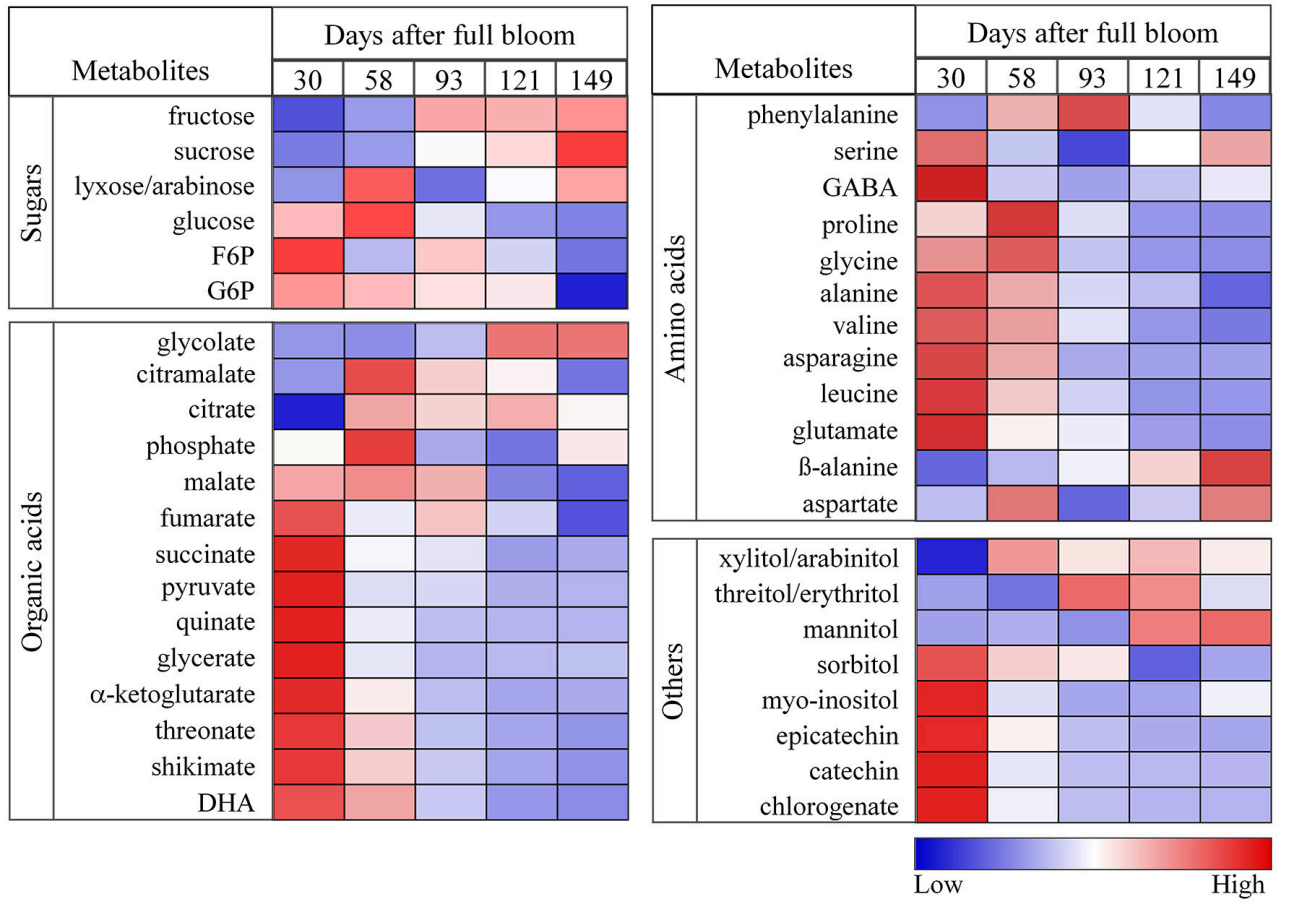
Heat maps showing the changes in the concentrations of sugars, organic acids, amino acids, sugar alcohols, and polyphenols during fruit growth (30, 58, 93, 121, and 149 days after full bloom). The deep blue color (low) denoted lower concentration of metabolites whereas the deep red color (high) denoted higher concentration of metabolites. The colors were generated from GC-MS metabolite profiling raw data (relative value) using the MultiExperiment Viewer software (MeV v4.9.0) after mean center and scale transformation into comparable levels.

To provide a more detailed representation of the results, heat maps were generated to show the metabolite changes during fruit growth (Figure 5). Fructose content increased significantly from 30 to 93 days and varied slightly later in development. The increase of fructose content was accompanied by a decrease of sorbitol level and starch degradation (Figure 2F) in the later growth stages. In contrast to the behavior observed in sorbitol, sucrose content increased from 30 to 149 days. Glucose reached its maximum level within 58 days and decreased throughout the later stages of fruit growth. G6P and F6P were high at 30 days and decreased gradually throughout fruit growth. Organic acids such as fumarate, succinate, pyruvate, quinate, glycerate, α-ketoglutarate, threonate, shikimate, and dehydroascorbate contents were high at 30 days and decreased substantially throughout fruit growth. In contrast, malate content increased up to 58 days and decreased considerably toward 149 days. Like most organic acids, the levels of most amino acids were very high in the early development and decreased gradually throughout fruit development. Most importantly, the major phenolic compounds present in apple (epicatechin, catechin, and chlorogenate) were very high at 30 days but strongly declined throughout fruit development (Figure 5, Figure S6C).

### Validation of isotopomers analysis

Fragments used for isotopomer analysis were selected by comparing the experimental mass isotopomer of tissue discs incubated with unlabeled substrate with the theoretically expected values (Table S2). The extent of incorporation of ^13^C-label into a certain metabolite was calculated using the molecular ion whenever available, otherwise, the average labeling of multiple fragments containing the carbon backbone of the molecular ion was considered. In some cases, when no suitable fragments could be found to represent the entire carbon skeleton, a single fragment was selected to represent the labeling of the metabolite in question (Roessner-Tunali et al., 2004). For example, *m/z* 218 of valine contains C1-C2, whereas *m/z* 144 contains C2-C5. However, *m/z* 144 seems to be superimposed with a minor peak from *m/z* 147, introducing about 9.3% error in m3, making its use for isotopomer analysis ambiguous (see Figure S4A). As a consequence, fragment *m/z* 218 was selected to represent the percentage labeling in valine. Similarly, fragment *m/z* 319 (C3-C6) of glucose was selected instead of taking into account *m/z* 160 that contains the first two carbons of glucose (C1-C2) because *m/z* 160 contains around 5% error (Figure S4B). In addition, as shown in Figure 3C, *m/z* 361 and 451 corresponding to the mass spectra of sucrose were selected to represent the glucosyl and fructosyl moieties of sucrose, respectively, using fragmentation simulation available in the NIST library. After the incubation of the tissue discs for 24 h with 20 mM [U-^13^C]glucose medium, a clear change in the mass isotopomer distributions of sucrose moieties were observed (Figure 3D). Koubaa et al. (2012) reported that the fructosyl moiety of sucrose contributes about 40% to the intensity of *m/z* 361, which, by the fragmentation simulation, is considered solely as the glucosyl moiety of sucrose. In contrast, the glucosyl moiety of sucrose contributes only a small amount (near 5%) to the intensity of *m/z* 451 (Koubaa et al., 2012), which implies that any dilution effect linked to the two moieties of sucrose could lead a different degree of labeling for each fragment. It is important to note that the fragments of sucrose at *m/z* 361, 437, and 451 gave the same isotopic enrichment (Table S2), as reported in previous studies (Alonso et al., 2005; Füzfai et al., 2008). In summary, we were able to estimate the natural stable istope abundance pattern in a range of fragments for each metabolite by selecting one or more representative fragments.

### Analysis of the ^13^C-label accumulation

**Figure 6:**
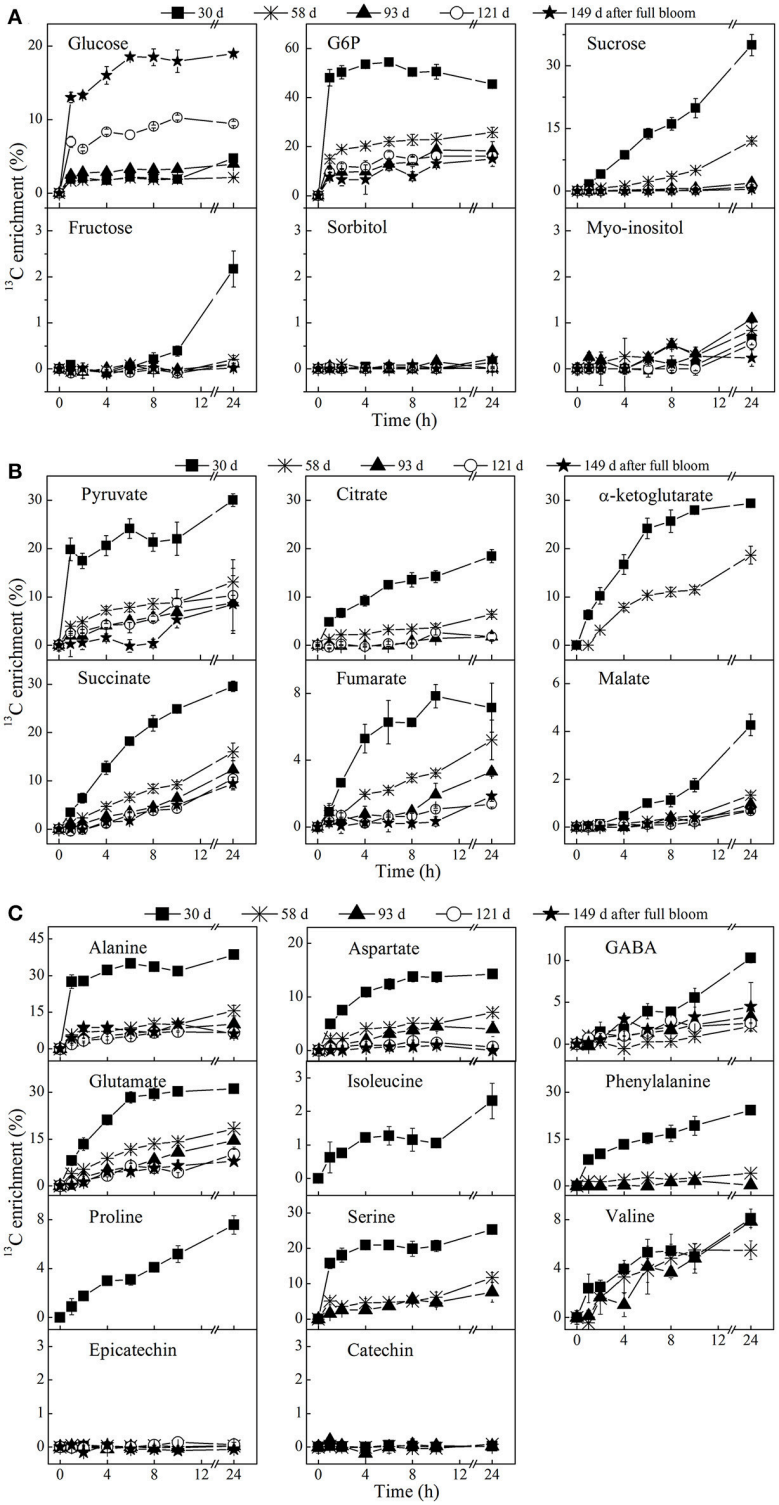
Time course of ^13^C-isotope enrichment of sugars **(A)**, organic acids (**B)**, and amino acid and polyphenols **(C)** following [U-^13^C]glucose loading. The enrichment values are different for each metabolite. The fruit were harvested at **five** growth stages, 30 days (■), 58 days (⋇), 93 days (▴), 121 days (◦), and 149 days (⋆) after full bloom and incubated in 20 mM [U-^13^C]glucose. Tissue discs were retrieved at 1, 2, 4, 6, 8, 10, and 24 h of incubation. The percentage labeling of isoleucine, proline, α-ketoglutarate, and phenylalanine were only calculated for the early growth stage(s), due to their very low concentrations as fruit growth advanced. Values are means of three independent replicates.

^13^C-isotope feeding experiments were performed to get a better insight into the metabolite changes and metabolic pathway activity during apple fruit development at five selected growth stages. Following the feeding of [U-^13^C]glucose, the ^13^C-label was incorporated into the various metabolites to different degrees of labeling depending on incubation time, metabolic pathway activity, and growth stage (Figure 6). The ^13^C-label was incorporated considerably into glycolysis intermediates 1 h after the addition of exogenous [U-^13^C]glucose. The labeling dynamics of metabolites in the glycolysis pathway was very fast and reached more or less a plateau within a few hours as indicated, for instance, by a nearly constant labeling of G6P (Figure 6A). Moreover, a higher percentage labeling of the majority of metabolites was observed at 30 days as compared to the later growth stages. In contrast, sorbitol, epicatechin, and catechin remained unlabeled during the 24 h incubation period, irrespective of the growth stages.

At 30 days, the enrichment of intracellular ^13^C-glucose reached 2.2% within 6 h and increased to 4.8% after 24 h while G6P and pyruvate ramped to 54.5 and 24.1% within 6 h, respectively. Between 6 and 24 h, the labeling of G6P slightly decreased while a continuous increase or nearly constant labeling was observed in the labeling dynamics of downstream metabolites. The labeling of pyruvate, citrate, succinate, malate, GABA, isoleucine, phenylalanine, proline, and valine increased over the time course of the feeding experiment. The labeling of α-ketoglutarate, alanine, aspartate, glutamate, and serine appeared to be approaching isotopic steady state between 10 and 24 h of the incubation. The incorporation of ^13^C-label into fructose and malate (the most abundant sugar and organic acid metabolite in apple, respectively) were quite similar and continuously increased during the time course of the experiment, except for fructose exhibiting a longer lag phase in ^13^C-label accumulation than malate. As compared to fructose, a much larger proportion of label was directed to sucrose that strongly increased throughout the feeding experiment, reaching over 35% within 24 h. Moreover, there was no considerable difference between the labeling pattern of glucosyl and fructosyl moieties of sucrose (Table 1).

**Table 1:** Percentage ^13^C enrichments of sucrose moiety following incubation in 20 mM [U-^13^C]glucose in apple tissue discs cut from 30 days (mean ± SE; n = 3).

**Incubation time (h)**	**Percentage** ^**13**^**C enrichment (sucrose moieties)**		**Mean difference**	***p*-value**
	**Glucosyl (*m/z* 361)**	**Fructosyl (*m/z* 451)**		
0	1.2 ± 0.1	1.0 ± 0.2	−0.4029	0.2018
1	3.2 ± 0.4	2.4 ± 0.1		
2	5.6 ± 0.9	4.7 ± 0.5		
4	9.6 ± 1.2	10.0 ± 0.7		
6	14.5 ± 2.1	15.1 ± 2.4		
8	17.0 ± 3.0	17.4 ± 2.2		
10	21.1 ± 4.3	20.8 ± 3.7		
24	37.4 ± 3.3	34.7 ± 5.5		

*The glucosyl (fragment m/z 361) and fructosyl (fragment m/z 451) moieties were used for the calculation of ^13^C-label enrichment, the average labeling reflects the labeling state of sucrose. Based on a paired t-test identifying the difference in labeling between the two sucrose moieties, the overall difference of the mean and the corresponding p-value was calculated*.

In the later growth stages (58–149 days), there was a much larger increase in the labeling of glucose in comparison with 30 days. The opposite was true for most of other metabolites, showing a decrease in percentage labeling throughout fruit development. It should be noted that GABA behaves differently. A decent amount of labeling of sucrose also took place at 58 days, exhibiting a gradual decrease in percentage labeling, whereas no considerable labeling of fructose could be found in the later growth stages.

#### Net ^13^C-incorporation in contrast with unlabeled pools

**Figure 7:**
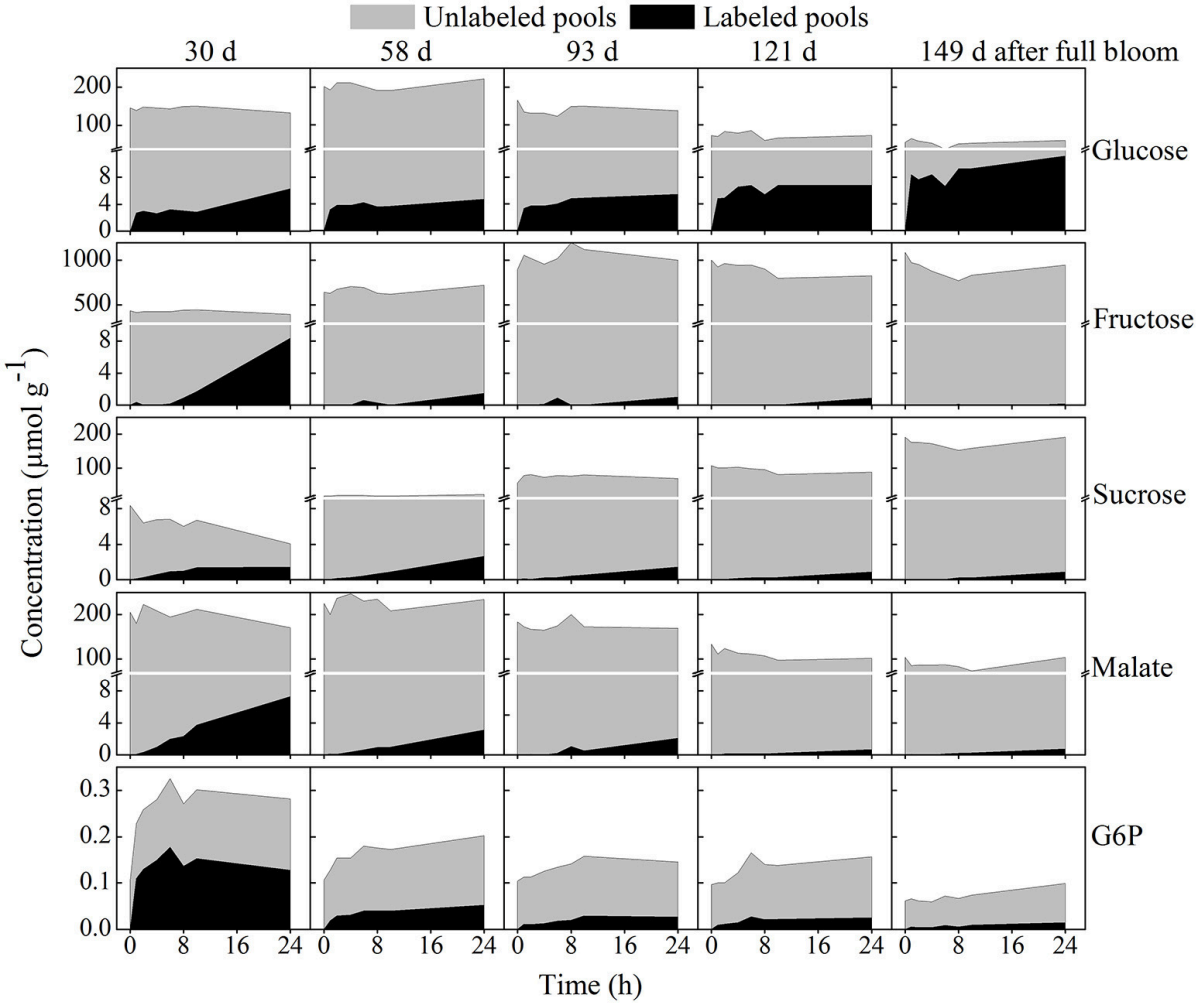
Time course of net ^13^C-incorporation in selected metabolites contrasted with unlabeled pools. The labeled pools are represented by the black area whereas the unlabeled pools are represented by the gray area. The five growth stages are ordered horizontally and similar scales were used for each metabolite across the five growth stages. Tissue discs were retrieved from five growth stages and incubated in 20 mM [U-^13^C]glucose for 24 h. Values are means of three independent replicates.

Figure 7 shows the total ^13^C-label incorporated into selected metabolites in comparison to the unlabeled pools of tissue discs retrieved from five growth stages during the feeding experiment with 20 mM [U-^13^C]glucose. Interestingly, the net ^13^C-incorporation into sucrose at 30 days reached a nearly constant value within a few hours (Figure 7) whilst the percentage ^13^C enrichment strongly increased, reaching over 35% within 24 h (Figure 6A) as the pool size of sucrose was reduced (from 8.4 μmol g^−1^ at time zero to 4.02 μmol g^−1^ at 24 h). The percentage ^13^C labeling of sucrose at 58 days was 11.9% at 24 h, however, the net ^13^C-incorporation (2.64 μmol g^−1^) showed a 2 fold increase relative to the early growth stage (1.4 μmol g^−1^; Table 2). This is because the total sucrose pool was smaller at 30 days (4.02 μmol g^−1^) than 58 days (22 μmol g^−1^).

**Table 2:** Total isotope accumulating into selected metabolites of apple tissue discs retrieved from five growth stages and incubated in 20 mM [U-^13^C]glucose for 24 h, at which most of the metabolites are reaching isotopic steady state.

**Metabolites (nmol g^−1^)**	**Days after full bloom**				
	**30**	**58**	**93**	**121**	**149**
**SUGARS AND SUGAR PHOSPHATE**					
Glucose	6, 315 ± 12.3	4, 848 ± 23.8	5, 461 ± 3.08	6, 816 ± 7.97	11, 204 ± 32.4
Fructose	8, 372 ± 91.8	1, 457 ± 58.1	1, 041 ± 82.4	891 ± 13.9	176.7 ± 81.1
Sucrose	1, 403 ± 8.14	2, 638 ± 18.3	1, 414 ± 10.1	868 ± 4.87	882.2 ± 72.6
G6P	127 ± 0.13	51.9 ± 0.25	26.5 ± 0.1	25.3 ± 0.22	14.7 ± 0.39
**ORGANIC ACIDS**					
Pyruvate	34.4 ± 0.22	6.91 ± 0.13	10.3 ± 0.12	12.0 ± 1.72	8.1 ± 1.04
Citrate	55.8 ± 0.05	30.1 ± 0.09	8.95 ± 0.31	8.9 ± 0.27	nd
α-ketoglutarate	62.1 ± 0.18	15.7 ± 0.11	Nd	nd	nd
Succinate	69.5 ± 0.60	17.1 ± 0.18	19.8 ± 0.85	17.4 ± 0.25	26.8 ± 0.99
Fumarate	4.6 ± 0.00	4.4 ± 0.02	2.71 ± 0.03	2.1 ± 0.02	3.3 ± 0.00
Malate	7, 250 ± 191	3, 117 ± 33.3	1, 636 ± 16.8	689 ± 6.7	736 ± 25.0
**AMINO ACIDS**					
Alanine	654 ± 2.48	110 ± 3.31	56.1 ± 3.28	7.2 ± 0.02	23.1 ± 4.53
Valine	12.7 ± 0.15	7.23 ± 0.02	8.34 ± 0.03	nd	nd
Serine	51.4 ± 0.10	11.5 ± 0.47	3.92 ± 0.45	nd	nd
GABA	3.50 ± 0.05	0.59 ± 0.01	1.09 ± 0.08	0.5 ± 0.07	1.6 ± 0.7
Aspartate	321 ± 0.79	113 ± 4.66	31.1 ± 5.48	1.66 ± 0.82	nd

*Values are means ± SE (n = 3). nd, not detected, due to very low metabolite concentrations it was not possible to calculate reliably the relative distribution of mass isotopomers both in unlabeled and labeled ions*.

Unlike most other metabolites, the total isotope accumulation in intracellular glucose was much more prominent in the later growth stage. For instance, after 24 h of incubation the absolute amount of the total isotope incorporated at 30 days in glucose reached 6.31 μmol g^−1^ whereas the value was considerably higher in the later growth stage (149 days), reaching 11.2 μmol g^−1^ although the total concentration of glucose was decreased at the later growth stage (Figure 7). Fructose labeling was high in young fruit and decreased over the growth stage. In addition, more label was incorporated into malate than in all other organic acids combined (Table 2).

## Discussion

### Rationale for developing ^13^C-labeling experimental setup for developing apple fruit

In developing apple fruit, sorbitol and sucrose enter the fruit respiratory metabolism (Berüter et al., 1997) predominantly through the apoplastic pathway (Zhang et al., 2004), essentially after being converted into free fructose and glucose (Loescher et al., 1982; Büttner and Sauer, 2000; Williams et al., 2000). Several studies have been published using labeling with [U-^13^C]glucose to study the central metabolic fluxes in many plant species (apple, Berüter, 2004; potato tubers, Roessner-Tunali et al., 2004; maize root tips, Alonso et al., 2007). To establish incorporation of ^13^C labeled sugars within the central metabolism, we allowed tissue discs cut from growing fruit to take up uniformly ^13^C labeled extracellular glucose from the medium. We therefore carried out an additional set of experiments to establish the rate of glucose uptake and the labeling kinetics of the various metabolite pools. The preliminary experiment was performed at three distinct growth stages: i.e., 30 days (cell division), 90 days (cell expansion), and 150 days (maturation) throughout the 2014–2015 growing season. These preliminary results indicated a fast labeling of the upstream metabolites resulting in saturating of the labeling within a few hours of incubation. The ^13^C-label was incorporated into the various metabolites in just 1 h after the addition of labeled substrate. This rate of incorporation is similar to what was observed for the accumulation of [^14^C]sorbitol in apple tissue discs (Zhang et al., 2004) but very fast when compared with the uptake of carboxyfluorescein (a common fluorescent marker of phloem transport) supplied to the intact apple fruit pedicel, which takes up to 4 h to reach the fruit flesh (Zhang et al., 2004). It must be pointed out that in the current experiments [U-^13^C]glucose was supplied directly to tissue discs being a simplified model system whereas Zhang et al. (2004) supplied the carboxyfluorescein to the intact apple fruit pedicel so that it covered a longer transport distance to the fruit flesh.

An additional experiment showed that the incorporation of the ^13^C-label into the various metabolites increased linearly with the concentration of [U-^13^C]glucose in the range of 5–20 mM (Figure S3). This is in good agreement with the relationship between the source leave and sink fruit reported previously (Paul and Foyer, 2001; Morandi et al., 2008; Dash et al., 2013; White et al., 2015). These authors indicated that growth may be controlled by source and sink strength. For example, increasing carbohydrate availability through photosynthesis can increase growth to a certain extent. Also for sorbitol it has been demonstrated that its uptake by isolated tissue discs and in intact apple fruit is linear with respect to concentration (Berüter and Studer-Feusi, 1995). The final experiments were conducted using 20 mM [U-^13^C]glucose to ensure excess amount of label in the medium to allow incorporation of decent amounts of label in a broad range of metabolites (see “Materials and Methods”). In addition, the osmotic strength of the medium was adjusted to the osmolality of the different growth stages in the range of 228–790 mOsmol kg^−1^ by using betaine (Figure 2A) – thus to preserve the integrity of tissue discs submerged in liquid medium – whilst the glucose concentration was kept the same for the different growth stages.

### Dynamic labeling revealed metabolite features of developing apple fruit

#### Reduction in respiration rate during fruit growth can be related to changing label incorporation

Like pear fruit (Zhang et al., 2005), apple is characterized by high respiration rate at the early growth stage which then generally decreases throughout the season until prior to initiation of fruit ripening (Bepete and Lakso, 1997). During the early stage (30 days) when cell division was most active (Janssen et al., 2008), fruit showed the highest respiration rate (Figure 2D), driving utilization of the imported labeled glucose into the wider metabolism (Figure 6). Given the expected high FK/HK (fructose- and hexose-kinase) enzyme activities at the early growth stage of the fruit (Li et al., 2012), rapid utilization of the imported sugars for cell division and fruit growth is facilitated (Berüter, 1985; Dash et al., 2013). With fruit development progressing respiration rate gradually declined and most of the labeled glucose taken up was no longer transferred to the other metabolites (Figures 6, 7). This reduction might be associated with the decrease in HK activity during fruit growth (Li et al., 2012). Similarly, Beauvoit et al. (2014) reported a higher glycolytic flux during cell division and a sharp decline during cell expansion of tomato fruit.

In general, carbon accumulation in a sink fruit depends on sink characteristics such as uptake rate (sink size × sink activity), photosynthesis and respiration rate of the fruit, as well as acquisition of carbon via the peduncle (Henton et al., 1999; Paul and Foyer, 2001; White et al., 2015). As reported for tomato (Greve and Labavitch, 1991), the ^13^C-label incorporation into actively growing tissue is much higher than into fully grown yet ripening tomato pericarp. Therefore, this data confirms the tight relationship between the fruit's respiration rate and its glycolytic capacity, both changing during fruit development (Figure S5). Moreover, the declining net isotope accumulation into various metabolites observed at 58 and 93 days growth stages showed a slightly higher accumulation in some of TCA cycle metabolites such as fumarate, succinate, and malate at the later growth stage of 149 days (Table 2), which was in parallel with a slight increase in respiration and ethylene production rate (Figures 2D,E). In addition, the ^13^C-label incorporated into GABA was high at 149 days (1.5 μmol/g) as well as at 30 days (3 μmol/g), but lower at the intermediate growth stages (Table 2 and Figure 6C). Thus, the observed increase in label accumulation in TCA cycle metabolites, as well as the slight increase in the respiration and ethylene measurements at final growth stage reflects the onset of the climacteric rise (Dilley, 1981). This suggested that around 149 days after full bloom the “Braeburn” apple fruit were sufficiently mature for commercial harvest, at least for the concerning orchard and growth season.

The percentage ^13^C-label incorporated into glucose, especially in the early growth stages, was lower than the labeling of some other downstream intermediate metabolites such as G6P and pyruvate. This leads to the hypothesis that, early during development, tissue favors extracellular sugars over cellular sugars already stored in the vacuole as their main source of carbon, thus maximizing the fruit's net carbon loading. The fast glycolytic turnover of the imported labeled glucose only represents a small fraction against a large background of unlabeled glucose stored in the vacuole resulting in a low overall percentage of labeled glucose. This suggests a high carbon conversion efficiency in young fruit. The interfering effect of existing unlabeled pools of metabolites in interpreting labeling data has been recognized before (Alonso et al., 2005, 2007).

The pool of the glycolysis intermediate G6P was high at 30 days and decreased gradually throughout fruit development. The higher concentration of G6P early in development can be related to the higher respiratory flux in young fruits, resulting in a high incorporation of label into G6P, reaching over 54% (Figure 6A). Likewise, facilitated entry of radioactive glucose into the hexose phosphate pools were reported for tissue discs retrieved from 50% fully grown apple fruit (Berüter et al., 1997). G6P could have been incorporated into sucrose, starch and cell wall polysaccharides (Alonso et al., 2005), as well as into the glycolytic pathway as indicated by the massive ^13^C-label incorporation in the current experiments. G6P can also be used as a substrate for inositol synthesis in the cytosol, which can serve as a precursor for phytate synthesis (Mitsuhashi et al., 2008). During the cell expansion phase at 93 days, a slightly higher percentage labeling of myo-inositol was observed in the current study (Figure 6A) although it was evident that the net ^13^C-incorporation showed a higher value at 30 days relative to the later growth stage. The decreased G6P labeling between 6 and 24 h in young fruit is quite similar to that previously observed in potato tuber discs (Roessner-Tunali et al., 2004). The decrease could be related to either the turnover of unlabeled starch or the cycling of unlabeled sucrose. However, starch degradation mainly occurs in mature fruit (Janssen et al., 2008; Li et al., 2012) thus, the most likely explanation is the regeneration of G6P through sucrose cycling (Berüter et al., 1997; Beauvoit et al., 2014). In agreement with our hypothesis, a decreasing sucrose level was observed during the 24 h incubation at 30 days (Figure 7).

#### Sucrose cycles in growing fruit

As reported for potato tuber discs (Roessner-Tunali et al., 2004), a substantial labeling of sucrose took place during fruit development (Figure 6A). This suggests that in addition to the large flux of sucrose translocated from the leaves (Klages et al., 2001), the fruit is actively sustaining its sucrose synthesis apparatus, confirming that sucrose is the major product of sugar metabolism. Sucrose may be synthesized via conversion of F6P and uridine diphosphate-glucose (UDP-Glc) catalyzed by sucrose phosphate synthase (SPS) or from UDP-Glc and fructose as catalyzed by sucrose synthase (SuSy) which has higher activity in young fruit (Li et al., 2012) (see Figure 1). Given there was no significant difference between the labeling pattern of glucosyl and fructosyl moieties (Table 1), sucrose synthesis is most likely mediated by SPS with F6P inheriting the labeling directly from G6P through isomerization, suggesting that the hexose-phosphate isomerase reaction is at equilibrium. Alonso et al. (2005) demonstrated that the labeling state of the glucosyl and fructosyl moieties in isotopic steady state conditions reflects the labeling state of G6P and F6P, respectively. At the same time, fructose labeling (Figure 6A) exhibited a longer lag phase as compared to G6P and sucrose. If sucrose was coming from fructose and UDP-Glc via the SuSy mediated reaction, the fructose moiety of sucrose would have been diluted by unlabeled fructose, at least in the first few hours of incubation when fructose labeling was not initiated. This means that sucrose synthesis via SuSy would lead to a higher labeling of the glucosyl moiety of sucrose, when labeled glucose was supplied (Geigenberger and Stitt, 1993). Altogether, equal labeling of the two moieties of sucrose can only be achieved through SPS and therefore, sucrose synthesis was most likely mediated by SPS.

In young fruit at 30 days, the overall sucrose content decreased by twofold (from 8.4 to 4.01 μmol g^−1^) during 24 h of incubation. Its increased percentage labeling and its overall low and decreasing levels indicated sucrose was actively metabolized during early development in line with the high metabolic demand related to cell division and growth. Evidence for the rapid turnover of sucrose during the early growth stage of tomato fruit—after being transported into the vacuole, due to a higher vacuolar invertase (VI) activity—is reported by Beauvoit et al. (2014). Similarly, a high VI activity during the early growth stage of rapid cell division of apple and peach fruit has already been published (Li et al., 2012; Zhang et al., 2013). This implies that VI and SPS enzymes are recognized as the main players of sucrose cycling in many sink tissues.

At 58 days, where cell expansion rate reached its peak (Janssen et al., 2008; Li et al., 2012), there was a significant increase in total isotope accumulation in sucrose as compared to the early and the later growth stages (Table 2 and Figure 7). The two fold increase in net ^13^C-label incorporation into sucrose implies that ^13^C allocation into reserves was high during cell expansion with limited conversion to intermediate metabolites. It underlines the importance of sucrose cycling in controlling the glucose level in the cell, regulating the glycolytic capacity of plant cells. This increased labeling of sucrose can be related to the peaking glucose phosphorylation activity and the higher glucose content during this period of apple fruit development as observed by Zhao et al. (2016). In line with their report, a higher glucose content was observed at 58 days (Figure 5, Figure S6).

SPS is known to play an important role in this as well but shows various behaviors for the different fruits. In developing peach fruit SPS activity was reported to increase during the most rapid sucrose accumulation phase (Lowell et al., 1989; Zhang et al., 2013), which is consistent with the higher sucrose labeling observed at 58 days. In apple fruit, SPS activity was shown to increase slightly with progressing fruit growth followed by a rapid increase during ripening, mirroring the reduction in starch level (Berüter and Studer-Feusi, 1997; Li et al., 2012), which is inconsistent with the currently decline in labeling of sucrose toward maturation. Similarly, an increase in SPS activity during kiwifruit ripening was reported (Nardozza et al., 2013). This concurrent high SPS activity and low labeling of G6P and sucrose currently observed in mature fruit (Figure 6A) leads to the hypothesis that G6P is more likely to originate from starch degradation in the plastid than from exogenous [U-^13^C]glucose. Nardozza et al. (2013) observed a positive correlation between starch accumulation in high starch kiwifruit genotypes and SPS activity which leads to sucrose accumulation during ripening.

Furthermore, the incorporation of label into fructose can be explained from sucrose hydrolysis into fructose catalyzed by invertase and/or SuSy; particularly VI and SuSy are known to be more active in the early growth stage of some *Rosaceae* fruit such as peach (Lo Bianco et al., 1999) and apple (Li et al., 2012). The observed slower and lower labeling of fructose at 30 days can be explained from the much larger pool of unlabeled fructose (Figure 7). Such slow labeling of fructose was already found for *Arabidopsis* indicating that the hydrolysis of sucrose into glucose and fructose is evidently a slow process (Szecowka et al., 2013). Moreover, the absence of fructose labeling in young fruit during the first few hours of incubation suggests the glucose-fructose isomerase activity might be negligible with fructose being mainly formed from sucrose. Berüter (2004) reported a lower labeling of fructose as compared to the labeling of sucrose after addition of ^14^C-glucose. However, considering the absolute pool size of labeled carbon accumulated in fructose after 24 h (8.4 μmol g^−1^, Figure 7), which was higher than the pool of labeled sucrose, shows that the actual flux into fructose was very high. The decline in fructose labeling with fruit development (Figures 6A, 7) can be explained from the decreased activities of invertase and SuSy observed for apple (Li et al., 2012). In general, the labeling of fructose supports the hypothesis of sucrose cycling in apple (Berüter et al., 1997; Beauvoit et al., 2014). In contrast to sucrose and fructose, there was no ^13^C-label incorporated into sorbitol at any growth stage (Figure 6A) suggesting the conversion of sorbitol into fructose is effectively irreversible in developing apple fruit (Berüter et al., 1997).

Besides the gradual decline in percentage labeling, fructose and sucrose both increased in their concentration with fruit growth, becoming the most abundant soluble sugars in mature fruit (Figure S6). Fructose accumulation can be explained coming from either sorbitol or sucrose. Both sorbitol and sucrose are being transported from the leaves to the sink tissue and either are stored as such or converted into fructose (Loescher et al., 1982; Büttner and Sauer, 2000; Williams et al., 2000; Klages et al., 2001). The decrease of sorbitol levels during fruit growth can be related to the observed increase of fructose levels, which for sucrose was not the case. The increasing sucrose content with progressing fruit development can be associated with a decrease in sucrose-cleaving enzymes, invertase and SuSy in addition to an increase in sucrose synthesizing enzyme, SPS (Berüter and Studer-Feusi, 1997; Li et al., 2012). Another comprehensive example in addition to sucrose synthesis via SPS is starch degradation sustaining the energy used to fuel metabolic processes, hence, accumulation of incoming sucrose (Berüter, 2004; Nardozza et al., 2013; Mesa et al., 2016; Figure 2F).

#### Young fruits are characterized by high levels of organic and amino acids and polyphenols

Extensive metabolic changes occurred during fruit growth (Figure 5, Figure S6). Organic acids, free amino acids, and phenolic compounds exhibited a significant higher concentration in the early growth stage. These large pools of organic and amino acid metabolites can serve as building blocks for cell growth at the early growth stage. In addition to the larger pools of these metabolites, the higher accumulation of large amount of label in organic acids (Figure 6B) and amino acids (Figure 6C) observed in the early growth stage can be associated with a higher rate of glycolysis/TCA cycle metabolism and subsequent contribution to protein synthesis (Fernie et al., 2004a; Li et al., 2012) related to cell division. This allows the fruit to grow to a bigger extend (Farinati et al., 2017). The decrease in level and labeling with growth stage can be explained from the shift in processes from mainly cell division toward mainly cell expansion and maturation. Similarly, Ishihara et al. (2015) reported a higher rate of protein synthesis in young leaves characterized by a gradual decline with leave maturation.

The amount of label incorporated into malate was considerably higher than the total amount of label in the other TCA cycle intermediates (Table 2) which can be understood from the fact that malate is one of the major storage compounds in apple (Berüter et al., 1997). Interestingly, it should be noted that in all of the growth stages, fumarate showed a substantial increase in its concentration during the feeding experiments (see Figure S6B). Pyruvate and alanine showed a consistent pattern in their dynamics of label enrichment. The labeling of alanine was slightly higher than its precursor pyruvate. However, MS measurements may have been biased as consequence of very low pyruvate concentrations, as well as the high turnover rate of pyruvate and its localization in multiple subcellular compartments, with unlabeled pyruvate present in the various cellular compartments diluting the labeled pyruvate mainly present in the cytosol (Buescher et al., 2015). Isoleucine and valine, which are synthesized from pyruvate were strongly labeled. Aspartate and glutamate, which are formed by transamination reaction from oxaloacetate and α-ketoglutarate (Szecowka et al., 2013), also showed a more rapid ^13^C-label accumulation at 30 days.

The absence of label in epicatechin and catechin (Figure 6C) metabolites can be explained by their metabolic remoteness from the supplied ^13^C substrate although the label, to a certain level, did reach phenylalanine, which serves as a bridge between the plant primary metabolism and the polyphenolic pathway. Like most organic acids in the TCA cycle, chlorogenate, catechin, and epicatechin were synthesized, to a large extent, early during development (30 days) and gradually decreased during fruit growth (Figure 5). However, the expression level of anthocyanidin reductase and leucoanthocyanidin reductase, which are responsible for the synthesis of catechin and epicatechin in apple, are known to increase toward maturity (Henry-Kirk et al., 2012). Previous studies have suggested that the decline in the content of phenolic compounds is associated with a dilution effect linked to fruit growth (vacuole expansion in mature apple fruit) (Renard et al., 2007). In general, the changes of metabolite levels were consistent with previous studies of apple fruit growth (Zhang et al., 2010; Li et al., 2013).

In conclusion, the novelty of the present work is in the dynamic labeling experiments performed at various stages of fruit growth to study carbon re-allocation metabolism during apple fruit development. Interestingly, short time isotope feeding experiments showed a wide range of label distributions between the different growth stages, depending on the proximity of each metabolite to the substrate. Isotopic steady state labeling was achieved in the majority of metabolites within few hours of exogenous [U-^13^C]glucose addition. It is important to remark that young fruit is characterized by a greater degree of label accumulation, related to the higher metabolite demand during cell division and fruit growth. Due to the reduced metabolic activity, as mirrored by respiratory rate, ^13^C re-allocation into various metabolites gradually declined as the requirement for cell growth and carbon skeletons decrease with fruit development. The work presented here can serve as a platform for further studies to understand developmental changes associated with fruit growth. Positional isotope feeding experiments and metabolic modeling can be considered to furthermore quantify fluxes through the glycolysis and the pentose phosphate pathway.

## Author contributions

WB, MH, AG, and BN designed the experiments. WB carried out the experiments, data analysis and prepared the figures. MH, AG, and BN guided the experimental work. WB interpreted the results and wrote the manuscript with contributions from all the authors (VM, MH, AG, WVdE, and BN).

### Conflict of interest statement

The authors declare that the research was conducted in the absence of any commercial or financial relationships that could be construed as a potential conflict of interest.
