# Supplementary material for: Dynamic Labeling Reveals Temporal Changes in Carbon Re-Allocation within the Central Metabolism of Developing Apple Fruit
Source: Front Plant Sci. 2017 Oct 18;8:1785. doi: 10.3389/fpls.2017.01785 (PMC5651688; doi:10.3389/fpls.2017.01785)
Supplement: Supplementary file 1 [file Image1.PDF]

Supplementary Material

Dynamic labeling reveals temporal changes in carbon re-allocation in sink and central metabolites of apple fruit development

Wasiye F. Beshir[1], Victor B.M. Mbong[1], Maarten L.A.T.M. Hertog[1], Annemie H. Geeraerd[1], Wim Van den Ende[2], Bart M. Nicolaï[1,3]*

*** Correspondence:** Prof. Bart Nicolai: bart.nicolai@kuleuven.be

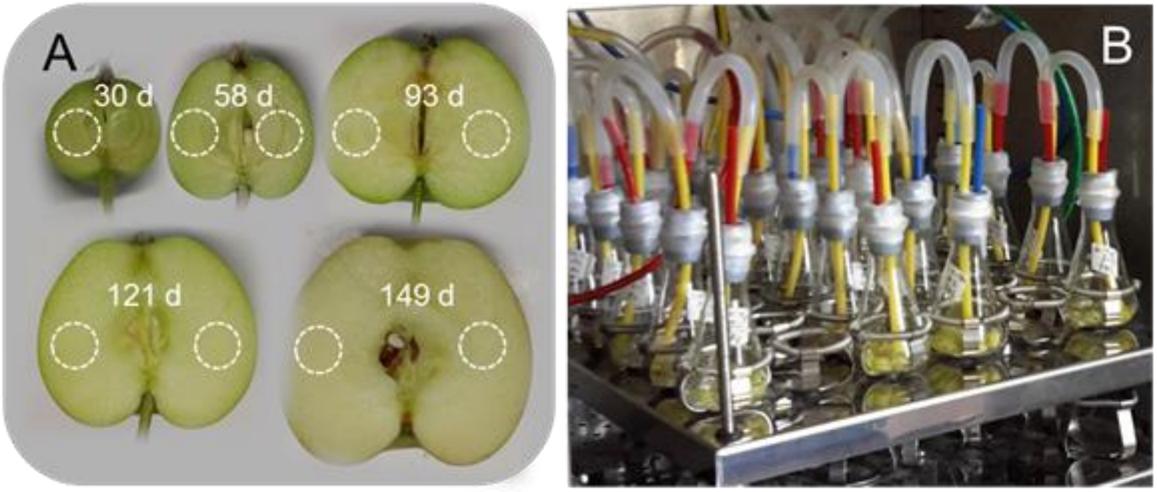

Figure S1. (A) Apple fruit at different stages of development (30, 58, 93, 121, and 149 d after full bloom with 24, 41, 55, 63, and 72 mm fruit diameter, respectively) considering the timing of major physiological events described in the text. The circle marked on the fruit indicating the positions were discs were recovered for the ^{13}C labeling experiments. (B) shows the *in vivo* ^{13}C-isotope feeding experiments conducted using the resulting apple tissue discs submerged in a liquid medium supplemented with 20 mM [U-^{13}C]glucose.
