# Supplementary material for: Dynamic Labeling Reveals Temporal Changes in Carbon Re-Allocation within the Central Metabolism of Developing Apple Fruit
Source: Front Plant Sci. 2017 Oct 18;8:1785. doi: 10.3389/fpls.2017.01785 (PMC5651688; doi:10.3389/fpls.2017.01785)
Supplement: Supplementary file 2 [file Image2.PDF]

Supplementary Material

Dynamic labeling reveals temporal changes in carbon re-allocation in sink and central metabolites of apple fruit development

Wasiye F. Beshir[1], Victor B.M. Mbong[1], Maarten L.A.T.M. Hertog[1], Annemie H. Geeraerd[1], Wim Van den Ende[2], Bart M. Nicolaï[1,3]*: *

Correspondence: Prof. Bart Nicolai: bart.nicolai@kuleuven.be

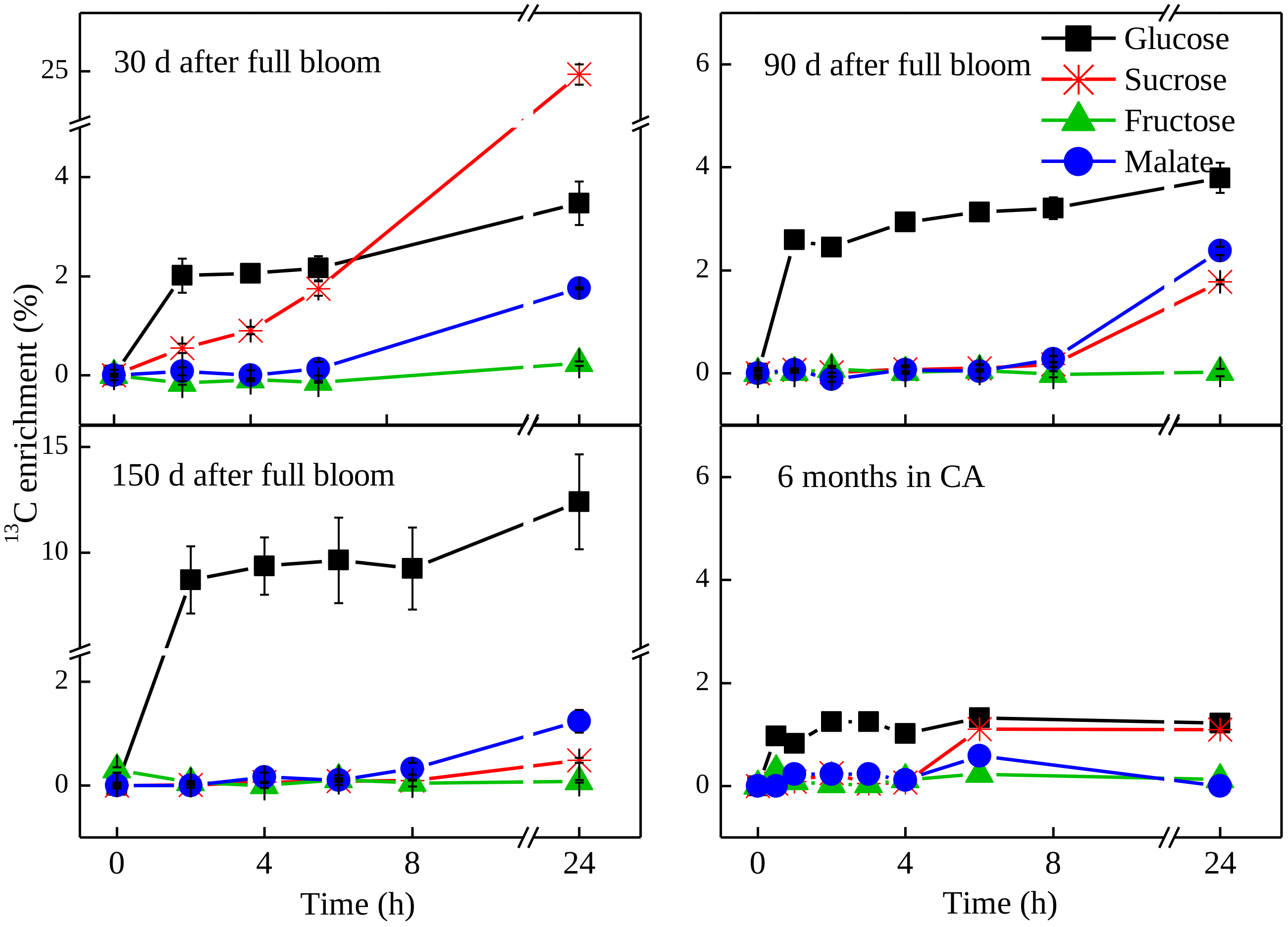

Figure S2. ^{13}C labeling kinetics of selected metabolites for fruit harvested at three distinct growth stages (30 d, 90 d, and 150 d after full bloom) and fully mature fruit (stored under commercial controlled atmosphere (CA) condition for six months. Tissue discs retrieved from “Braeburn” fruit were incubated in 20 mM [U-^{13}C]glucose for different time intervals. Percentage ^{13}C enrichment was determined after GC-MS analysis as demonstrated in the “Materials and Methods”.
