# Supplementary material for: Dynamic Labeling Reveals Temporal Changes in Carbon Re-Allocation within the Central Metabolism of Developing Apple Fruit
Source: Front Plant Sci. 2017 Oct 18;8:1785. doi: 10.3389/fpls.2017.01785 (PMC5651688; doi:10.3389/fpls.2017.01785)
Supplement: Supplementary file 3 [file Image3.PDF]

Supplementary Material

Dynamic labeling reveals temporal changes in carbon re-allocation in sink and central metabolites of apple fruit development

Wasiye F. Beshir[1], Victor B.M. Mbong[1], Maarten L.A.T.M. Hertog[1], Annemie H. Geeraerd[1], Wim Van den Ende[2], Bart M. Nicolaï[1,3]*

*** Correspondence:** Prof. Bart Nicolai: bart.nicolai@kuleuven.be

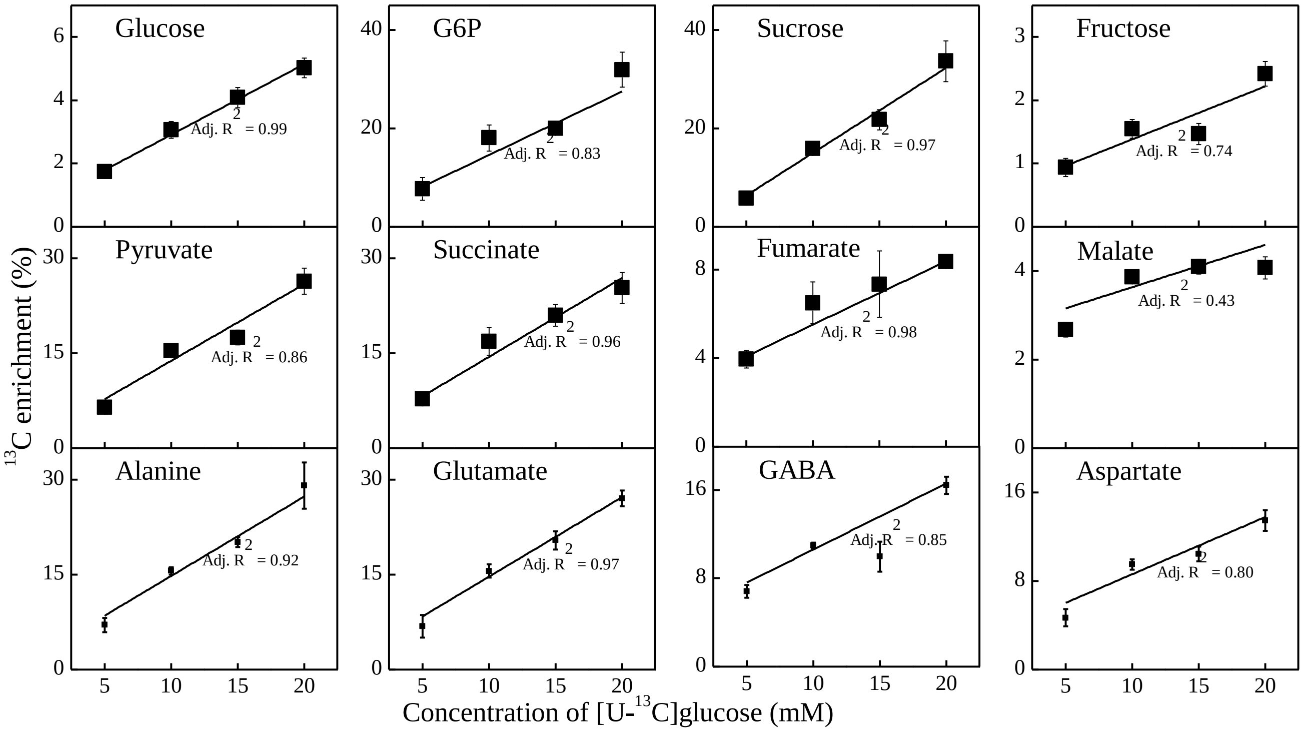

Figure S3. The relationship between the ^{13}C enrichment into the various metabolites and the concentration of [U-^{13}C]glucose in the medium, range of 5–20 mM. Tissue discs used for this glucose-fed test experiment were derived from 15 days after full bloom and incubated for 24 h after label introduction. Values are means ± SE (n = 3).
