# Supplementary material for: Dynamic Labeling Reveals Temporal Changes in Carbon Re-Allocation within the Central Metabolism of Developing Apple Fruit
Source: Front Plant Sci. 2017 Oct 18;8:1785. doi: 10.3389/fpls.2017.01785 (PMC5651688; doi:10.3389/fpls.2017.01785)
Supplement: Supplementary file 4 [file Image4.PDF]

Supplementary Material

Dynamic labeling reveals temporal changes in carbon re-allocation in sink and central metabolites of apple fruit development

Wasiye F. Beshir[1], Victor B.M. Mbong[1], Maarten L.A.T.M. Hertog[1], Annemie H. Geeraerd[1], Wim Van den Ende[2], Bart M. Nicolaï[1,3]*:

*** Correspondence:** Prof. Bart M. Nicolaï: bart.nicolai@kuleuven.be

(A) Valine (2TMS)

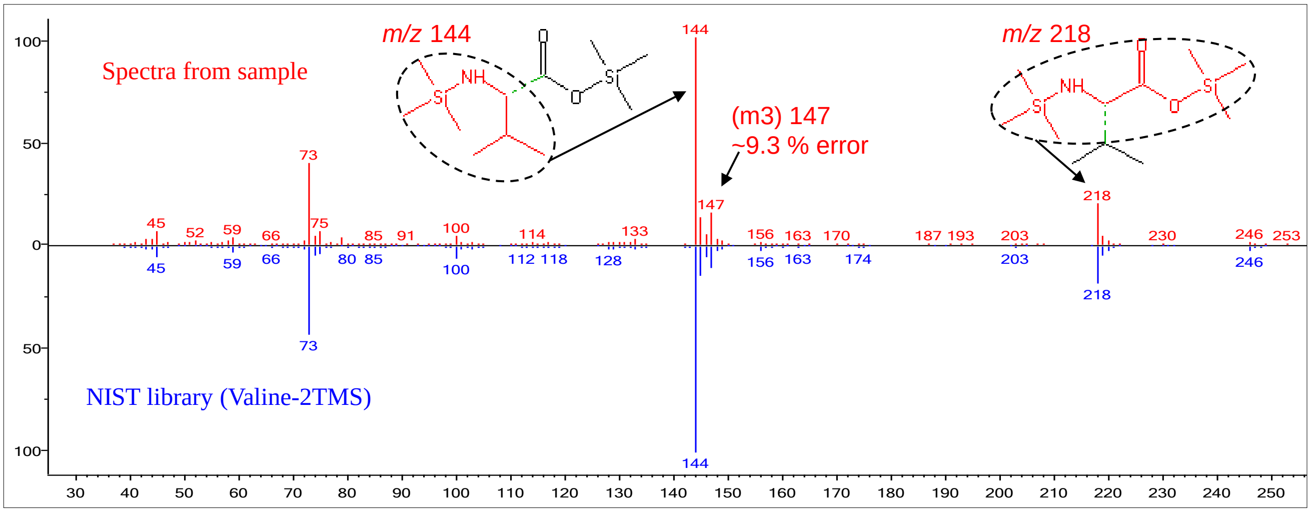

Fragment [f144]$^+$				Experimental values		
Fragment contains C	*m/z*	Mass isotopomers	$n^{13}C$ atoms	Relative isotopomer abundance (m_n)	Stdev	$m_n * n^{13}C$ atoms
C2-C5	144	*m0*	0	0.8366	0.0089	0.0000
	145	*m1*	1	0.0389	0.0016	0.0389
	146	*m2*	2	0.0061	0.0005	0.0121
	147	*m3*	3	0.1101	0.0080	0.3302
	148	*m4*	4	0.0084	0.0007	0.0335
			^{13}C enrichment (%)			10.37

(B) Glucose (1MEOX, 5TMS)

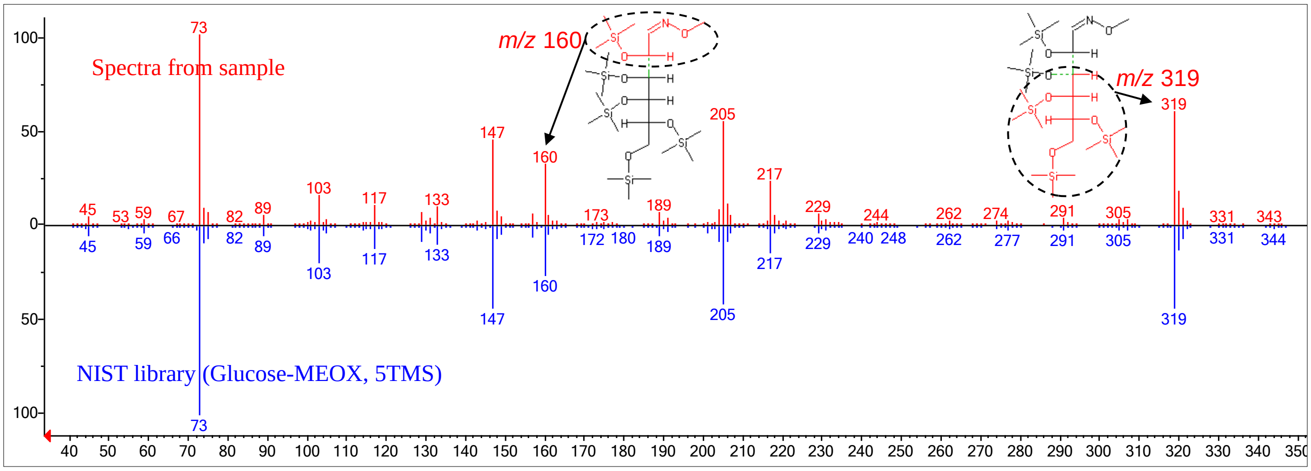

Fragment contains C	Fragment [f160]⁺ *m/z*	Mass isotopomers	$n^{13}C$ atoms	Experimental values: Relative isotopomer abundance (m_n)	Stdev	$m_n * n^{13}C$ atoms
C1-C2	160	*m0*	0	0.8924	0.0045	0.0000
	161	*m1*	1	0.0679	0.0018	0.0679
	162	*m2*	2	0.0024	0.0001	0.0049
	163	*m3*	3	0.0372	0.0029	0.1117
	13C enrichment (%)					6.15

Figure S4. GC-MS signal obtained for (MEOX-) TMS derivatized valine (A) and glucose (B) from tissue discs incubated with unlabeled substrate (red) in comparison with the NIST library (blue). Peak 144 of valine seems to be superimposed with a minor peak at 147, introducing about 9.3 % error in m3, making its use for isotopomer analysis ambiguous (A). Similarly, peak 160 of glucose contains a minor peak at 163 that introduce an estimated error of around 5 % (B).
