# Supplementary material for: Dynamic Labeling Reveals Temporal Changes in Carbon Re-Allocation within the Central Metabolism of Developing Apple Fruit
Source: Front Plant Sci. 2017 Oct 18;8:1785. doi: 10.3389/fpls.2017.01785 (PMC5651688; doi:10.3389/fpls.2017.01785)
Supplement: Supplementary file 5 [file Image5.PDF]

Supplementary Material

Dynamic labeling reveals temporal changes in carbon re-allocation in sink and central metabolites of apple fruit development

Wasiye F. Beshir[1], Victor B.M. Mbong[1], Maarten L.A.T.M. Hertog[1], Annemie H. Geeraerd[1], Wim Van den Ende[2], Bart M. Nicolaï[1,3]*

*** Correspondence:** Prof. Bart Nicolai: bart.nicolai@kuleuven.be

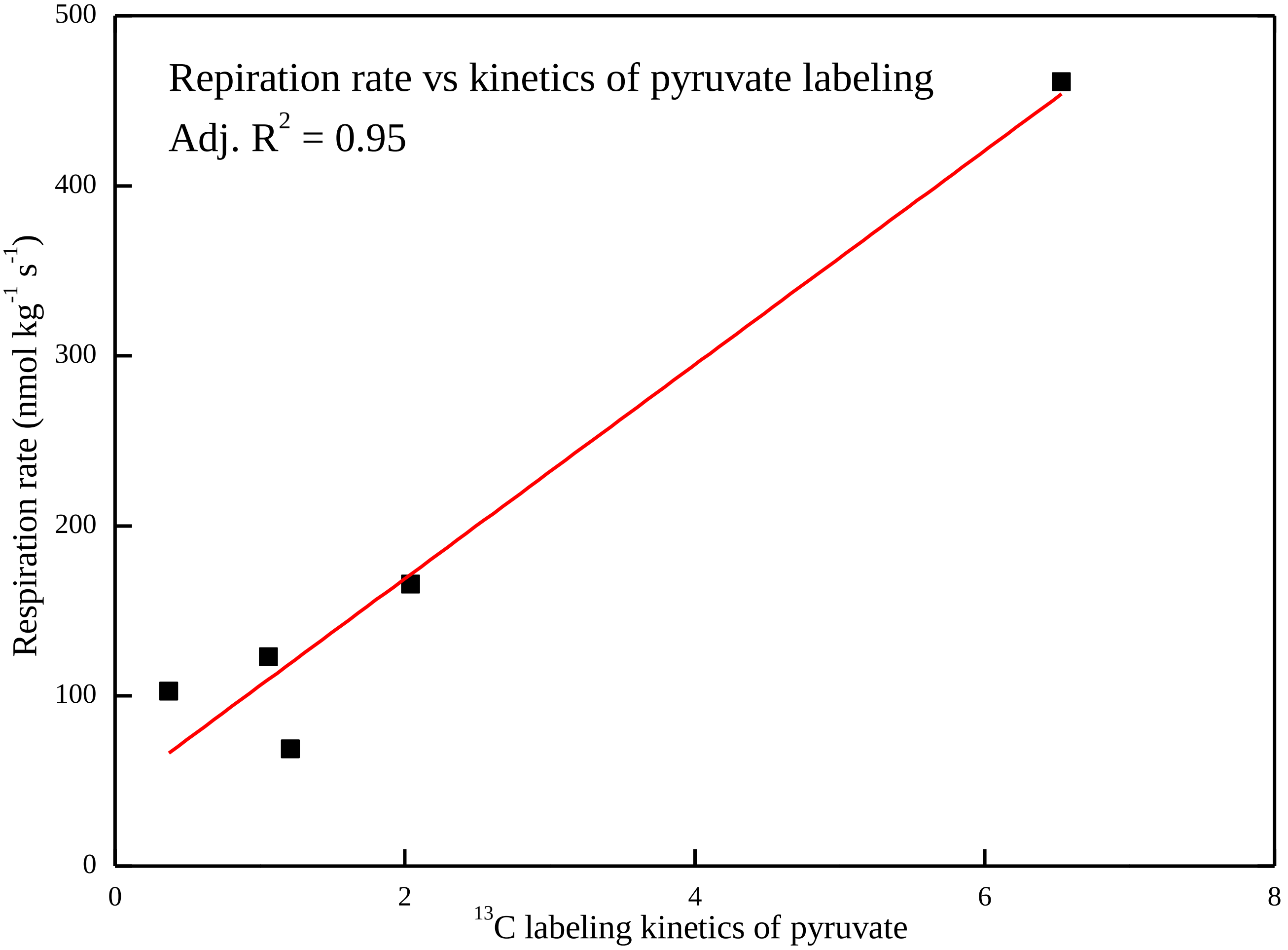

Figure S5. Fruit respiration rate (nmol kg^{-1} s^{-1}) as a function of ^{13}C labeling kinetics of pyruvate (assuming that pyruvate can reflect the fruit's glycolytic capacity) during fruit development. The slopes in the dynamic labeling phase of pyruvate (the first four hours of label incorporation) were used as ^{13}C labeling kinetics of pyruvate.
