# Supplementary material for: Dynamic Labeling Reveals Temporal Changes in Carbon Re-Allocation within the Central Metabolism of Developing Apple Fruit
Source: Front Plant Sci. 2017 Oct 18;8:1785. doi: 10.3389/fpls.2017.01785 (PMC5651688; doi:10.3389/fpls.2017.01785)
Supplement: Supplementary file 6 [file Image6.PDF]

Supplementary Material

Dynamic labeling reveals temporal changes in carbon re-allocation in sink and central metabolites of apple fruit development

Wasiye F. Beshir[1], Victor B.M. Mbong[1], Maarten L.A.T.M. Hertog[1], Annemie H. Geeraerd[1], Wim Van den Ende[2], Bart M. Nicolaï[1,3]*: *Correspondence, bart.nicolai@kuleuven.be

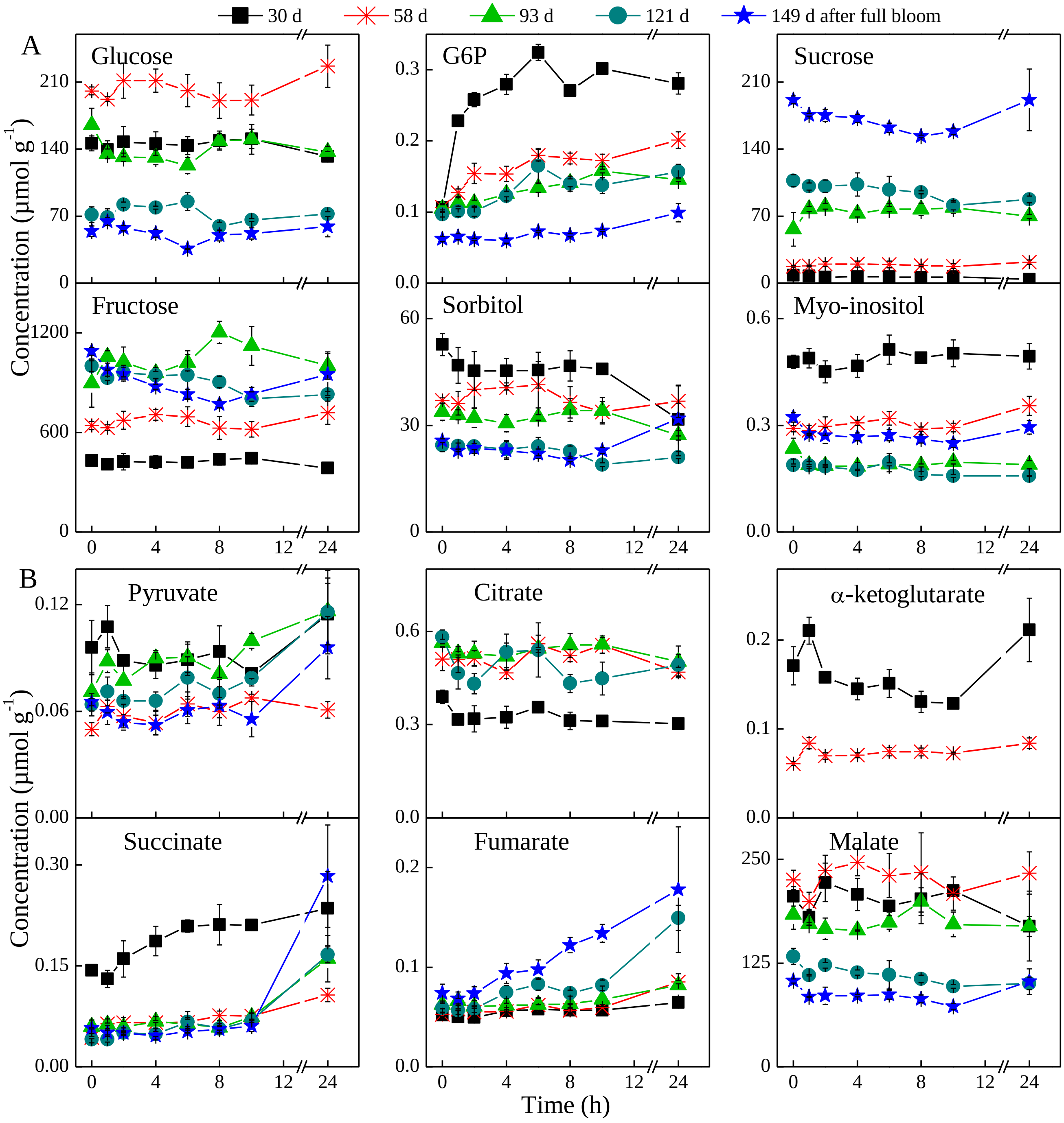

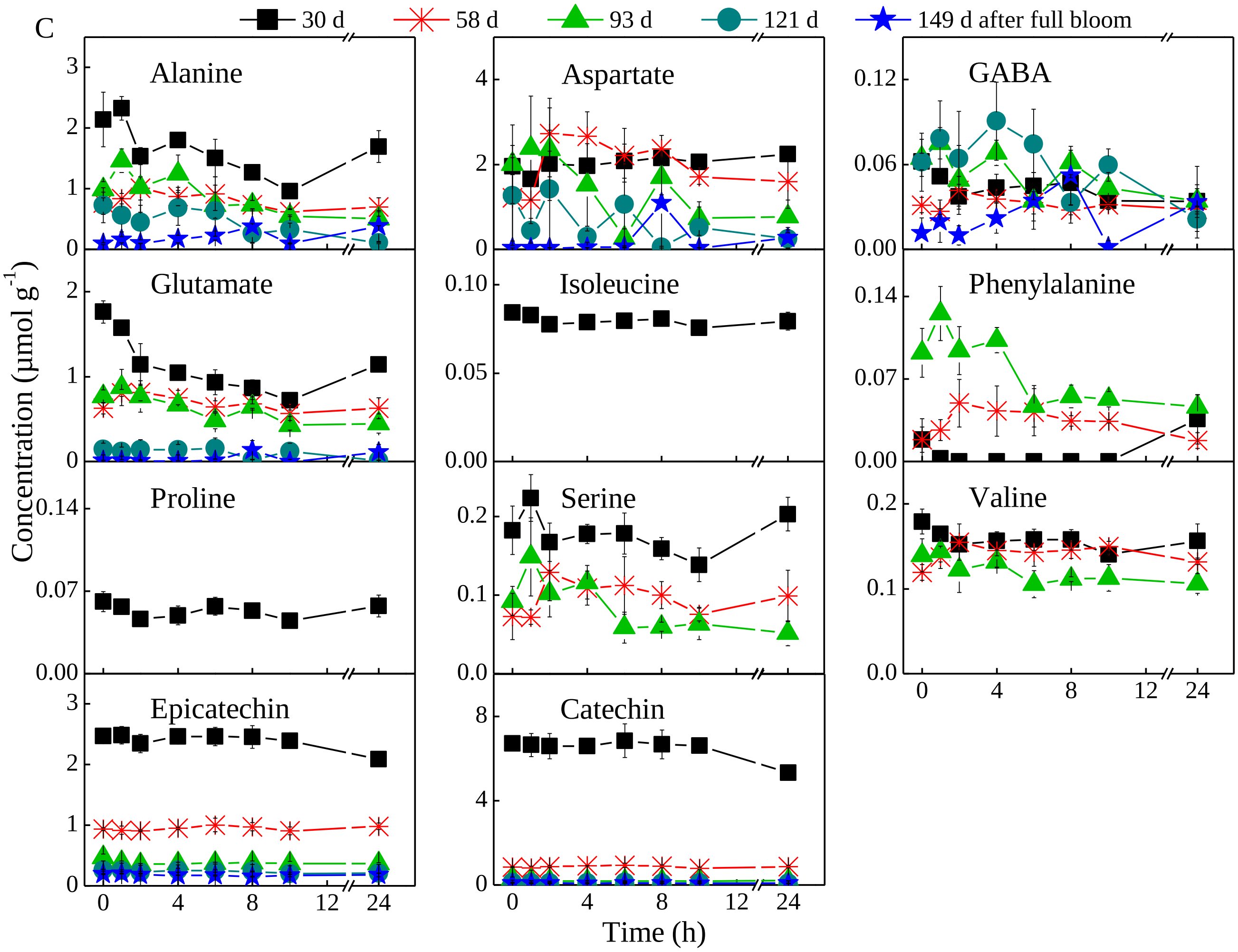

Figure S6. Absolute concentrations (μmol g^{-1}) of sugars (A), organic acids (B), and amino acid and polyphenols (C) shown in Figure 5, which were extracted from glucose-fed tissue discs derived from growing apple fruit. The fruit was harvested at five growth stages, 30 d (■), 58 d (∗), 93 d (▲), 121 d (●), and 149 d (★) after full bloom and incubated in 20 mM [U-^{13}C]glucose. Values are means ± SE (n = 3).
